# The complex neurochemistry of the cockroach antennal heart

**DOI:** 10.1007/s00441-024-03915-5

**Published:** 2024-09-06

**Authors:** Hans Agricola, Peter Bräunig

**Affiliations:** 1 Department of Cell Biology, Institute of Biochemistry and Biophysics, Center for Molecular Biomedicine, Friedrich-Schiller University Jena, Hans-Knoell-Strasse 2, 07745 Jena, Germany; 2 Department of Biology II (Zoology), RWTH Aachen University, Worringerweg 3, 52074 Aachen, Germany

**Keywords:** Insect, *Periplaneta americana*, Antenna, Neuromodulation, Neuropeptide, Octopamine, Allatostatin A, Head peptide, Proctolin, Serotonin, SNPF

## Abstract

The innervation of the antennal heart of the cockroach *Periplaneta americana* was studied with immunocytochemical techniques on both the light and electron microscopic levels. The antennal heart is innervated by two efferent systems, both using one biogenic amine in combination with neuropeptides. In one, we found co-localization of serotonin with proctolin and allatostatin. These fibers most likely originate from paired neurons located in the suboesophageal ganglion. In the second system, we found octopamine co-localized with the short neuropeptide F. The source of this second system is dorsal unpaired median (DUM) neurons, also located in the suboesophageal ganglion. The possible effects of these neuromediators on different targets are discussed.

**Supplementary Information:**

The online version contains supplementary material available at 10.1007/s00441-024-03915-5.

## Introduction

Apart from the main circulatory organ, the dorsal vessel (heart) in the abdomen, insects possess accessory pulsatile organs supplying appendages (Pass 1998, 2000; Hertel and Pass 2002). One of these, the antennal heart, pumps hemolymph into the antennae (Pass et al. 1988a; Wipfler et al. 2021; Kay et al. 2021). This organ was most thoroughly studied in cockroaches. Here, it supplies the antenna via vessels that run the entire length from the head capsule to the very tip of the flagellae. The vessels originate from paired elastic ampullae attached to the frontal head capsule near the base of the antenna. An unpaired muscle, the *Musculus interampullaris* (*M. interampullaris*), interconnects the two ampullae. In the sagittal plane, it is connected to the anterior end of the dorsal vessel, the aorta, by the paired strands of the *Musculus ampulloaorticus* (*M. ampulloaorticus*)*.* The *M. interampullaris* acts as a dilator, widening the lumen of the elastic ampullae during contraction to allow hemolymph to flow into them through their ostia. Once the muscle relaxes, the ostia closes, and hemolymph is pumped into the antennal vessels by the elastic force of the ampullae (Pass et al. 1988a; Wipfler et al. 2021). The antennal heart operates as a myogenic accessory circulatory organ with a beating rate of 27.3 ± 9.8 beats/min (Hertel et al. 1985). Its myogenic rhythm is modulated by neurons with axons running in the paired antennal heart nerves (*Nervus cardioantennalis,* a side branch of the *Nervus corporis cardiaci* III). Using cobalt iontophoresis, it was shown that these axons belong to neurons with somata located in the suboesophageal ganglion (Pass et al.1988a), a pair of lateral somata (ClP-neurons), and at least two dorsal somata belonging to unpaired neurons (DUM neurons). The axons of these neurons ascend the pharyngeal connectives to the corpora cardiaca, innervate the two muscles of the antennal heart, and terminate in a neurohemal tissue at the medial wall of the ampullae (Beattie 1976; Pass et al. 1988a). Using biochemical techniques, the biogenic amine octopamine (Pass et al. 1988b) and the neuropeptides allatostatin A, leucomyosuppressin, proctolin (Predel et al. 1999), and head peptide (Predel 2001) were detected in this system. Octopamine-immunoreactive innervation was demonstrated for the antennal heart of the locust *Schistocerca gregaria* (Antemann et al. 2018), but not yet for cockroaches. Allatostatin A immunoreactivity has been demonstrated in the antennal heart of the cockroach *Diploptera punctata* (Woodhead et al. 1992) and proctolin immunoreactivity (PROC-ir) in that of *Periplaneta americana* (Hertel et al. 2012). The physiological effects of allatostatin A or of proctolin were comparable in both species (Hertel et al. 1988, 1995; Hertel and Penzlin 1992; Lange et al. 1993). Proctolin had very potent myostimulatory effects; allatostatin A, in contrast, had no effect on the muscles (Lange et al. 1993). The action of octopamine was described as inhibitory, and leucomyosuppressin elicited a complete block of muscle activity even at low concentrations (10^−8^ M; Hertel 1994).

Although there is already extensive knowledge about the neurochemistry of the antennal heart, several questions remain unanswered. How are these different neuromediators distributed among the two different types of neurons previously identified? Are some of these mediators co-localized in single neurons, perhaps even in the same vesicular structures? Are there differences in the innervation pattern of the substructures of the antennal heart, such as the muscles, the corpora cardiaca, the ampullae, or the neurohemal tissue of the ampullae? Finally, are there additional mediators that might influence the antennal heart? In order to answer these questions, we tested seventeen different antisera or monoclonal antibodies from rabbits or mice against GABA, biogenic amines, and neuropeptides (Table 1). As will be shown here, using immunofluorescence microscopy and the pre- and post-embedding techniques for transmission immunoelectron microscopy, only five of the seventeen antibodies yielded positive signals in the substructures of the antennal heart. The ultrastructural investigation revealed the co-localization of several neuromediators in single neurosecretory granules. The consequence of this observation is discussed, as are the possible influences of these five substances on the antennal heart itself and sensory epithelia of the antenna.

**Table 1:**
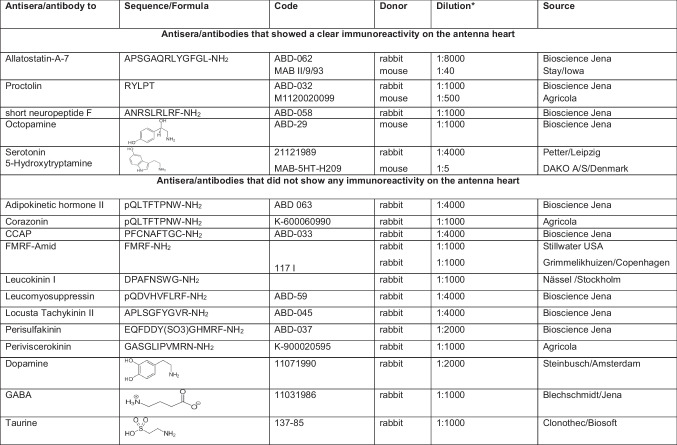
List of antisera/antibodies used in the present study

^*^For immunofluorescence

## Material and methods

### Animals

*P. Americana* L. (Blattodea) was taken from an established culture at the Zoological Institute of the Friedrich Schiller University. A total of 98 male cockroaches, at least 2 days after adult eclosion, were used in this study.

### Preparation

The insects were immobilized for 1 h in the refrigerator and subsequently decapitated. The head was mounted frontal side up in a Sylgard®-lined dish. A window was cut into the frontal cuticle to expose the antennal heart. A 4 °C cold fixative (see below) was applied as a drop onto the antennal heart. After 10 min, the antennal heart, corpora cardiaca, and/or suboesophageal ganglion were dissected and transferred into buffer-filled siliconized glass dishes and fixed at 4 °C in the refrigerator.

### Immunocytochemistry

For immunolabeling, we used four different protocols (described below), two for light microscopy (1L and 2L) and two for electron microscopy (1E and 2E). The co-localization experiments required further adjustments to these four protocols. Such modifications are documented in detail in Supplementary Table 1.

### Light microscopic immunolabeling of biogenic amines, amino acids, and the neuropeptide proctolin (protocol 1L)

For the detection of GABA, taurine, dopamine, octopamine, and tissues were placed in fixative (2% formaldehyde, 1% glutardialdehyde in 0.1 M sodium cacodylate) for 3 h and then rinsed in Tris buffer (TB) with sodium metabisulfite (TB-SMB; 0.5 M Tris, sodium metabisulfite at 8.5 g/l, pH 7.4) at room temperature. The following day, the antennal hearts were incubated for 10 min in TB-SMB with 0.1 M sodium borohydrite and washed five times for 15 min in TB-SMB. This was followed by incubation for 24 h with the primary antibody (for dilution, see Table 1) in a blocking solution at 4 °C (TB-SMB with 0.25% Triton X-100 (TX), 1% normal goat serum (NGS), 0.25% bovine serum albumin (BSA), 3% fat-free milk powder). The next day, tissues were rinsed three times at room temperature in Tris-buffered saline (0.5 M Tris, sodium chloride at 8.5 g/l, pH 7.5) and then incubated with the secondary antiserum for at least 12 h at 4 °C in the blocking solution. The following secondary antisera were used: goat antimouse IgG (GAM) tagged with either CY2 or CY3 (Dianova GmbH, Hamburg) and diluted at 1:200, or goat antirabbit IgG (GAR) tagged with either CY2 or CY3 (Dianova GmbH, Hamburg) diluted at 1:600.

### Light microscopic immunolabeling of all other neuropeptides (protocol 2L)

The antennal hearts were fixed in 4% formaldehyde in 0.1 M Millonig’s phosphate buffer (14.5 g Na_2_HPO_4_ × 2H_2_O, 2.62 g NaH_2_PO_4_ × H_2_O, 5 g NaCl in 1000 ml double-distilled (dd) H_2_O, pH 7.3). After extensive rinsing in buffer, incubation was carried out with the primary antibody (for dilutions, see Table 1) for 24 h in the blocking solution at 4 °C (10 ml of 0.1 M Millonig’s phosphate buffer, 300 mg fat-free milk powder, 25 mg BSA, 1% NGS, 0.5% TX). Following another extensive rinsing in buffer, the tissue was incubated for 24 h at 4 °C in one of the secondary antisera described above.

### Embedding, storage, and evaluation of preparations

The wholemounts were extensively rinsed in the appropriate buffer solutions, cleared in an ascending glycerol series (40%, 60%, 80%), mounted on gelatine-coated slides (1 g gelatine, 0.1 g chromium potassium (II) sulfate, and 100 ml ddH_2_O), and embedded in Mowiol (Sigma-Aldrich GmbH, Taufkirchen). They were stored in the refrigerator until further examination.

Results were documented with the camera of the light microscope (Zeiss-Axiophot) on Kodak Elite 400 film. Slides were digitized with a scanner (CanoScan 9000F Mark II) at 600 dpi. Alternatively, images were acquired using a Hamamatsu digital camera C4742-95 attached to the microscope and connected to a G4 Power Mac. The software OpenLab (Improvision Limited, Coventry, England) was used for image documentation. Image analysis was carried out with Adobe Photoshop CS5 Extended Version 12.0.4 × 64.

### Post-embedding immunogold method for electron microscopy

The starting point for electron microscopic immunocytochemistry was vibratome sections of the antennal heart. Wholemounts of the antennal heart were embedded in a block of 5% agarose at 60 °C and mounted on a holder. Subsequently, the agarose block and holder were fixed (see below) further overnight in the refrigerator. Using a homemade vibratome, 50-µm-thick sagittal and horizontal sections were obtained from the antennal heart, corpora cardiaca, or suboesophageal ganglion. The sections floating freely in TB-SMB were picked up with a brush and transferred into buffer-filled siliconized glass dishes.

#### Protocol 1E

Sections were fixed in 0.5% glutardialdehyde in 0.1 M HEPES buffer at pH 7.35 (11.9 g HEPES in 470 ml ddH_2_O and 1 N NaOH to adjust the pH to 7.35 and 500 ml ddH_2_O, adjusted to 380 mosm with sucrose), and, after intensive rinsing overnight, post-fixed in osmium tetroxide (see Supplementary Table 1).

#### Protocol 2E

Sections were fixed in 4% formaldehyde and 0.1% glutardialdehyde in 0.1 M HEPES buffer at pH 7.35, adjusted to 380 mosm with sucrose. After intensive rinsing overnight in buffer, some preparations were post-fixed with OsO_4_ (see Supplementary Table 1).

After fixation, specimens were rinsed in buffer, dehydrated in an ascending acetone series, and embedded in Durcupan ACM (FLUKA Chemie GmbH). Ultra-thin sections (around 70 nm) were cut with a Reichert Ultracut E-microtome and transferred to Formvar-coated nickel grids.

Grids were placed on drops containing the reagents and were continuously moved by self-rotation on a magnetic stirrer (nickel grids!). The immunogold incubation steps were performed in a moist chamber. Grids were first immersed in Tris buffer with Triton X-100 (TB-TX, 0.05 M Tris-buffered saline at pH 7.4 containing 0.1% Triton X-100) for 10 min. Three rinses in TB-TX followed. Next, grids were incubated in primary antibody solution (dilution 1:100 to 1:500) in TB-TX with NGS (1:20) for 4 h, followed by three rinses with TB-TX. Subsequently, grids were incubated for 2 h in secondary antibody solution (GAR or GAM conjugated to 6, 10, or 12 nm colloidal gold; Dianova GmbH, Hamburg), diluted 1:100 in TB-TX. Subsequently, they were rinsed in ddH_2_O, air dried, and, in some cases, contrasted with uranyl acetate for 15 min (Ude und Agricola 1995). As a control, a few grids from each batch were processed using a protocol where the primary antibody was replaced with normal rabbit serum. Ultrathin sections were evaluated on a Zeiss EM 900 transmission electron microscope.

### Pre-embedding PAP method for octopamine labeling for electron microscopy

Transversally cut 50-µm-thick vibratome sections were made from the dilator muscle of the antennal heart in cacodylate buffer (0.1 M cacodylate, SMB at 10 g/l, pH 6.2) and fixed for 3 h as described by Dacks et al. 2005 (0.1 M sodium cacodylate, 2% paraformaldehyde, 1% glutaraldehyde). Fixation was followed by three rinses in Tris buffer with SMB (0.5 M Tris, sodium metabisulfite at 8.5 g/l, pH 7.4). Sections were immersed in 10, 20, and finally 30% sucrose in buffer and transferred into a box made from aluminum foil. After removing the liquid, sections were briefly immersed in liquid nitrogen (− 196 °C) in order to create micropores in membrane structures for better penetration of the antisera. After this treatment, the sections were transferred to glass dishes, rinsed in TB-SMB three times for 15 min, and subsequently incubated for 24 h in a blocking solution (TB-SMB and 0.25% TX100, 1% NGS, 0.25% BSA, 3% fat-free milk powder, centrifuged for 10 min at 16,000 rpm) containing the monoclonal antibody against octopamine at a dilution of 1:500. After extensive rinsing in buffer, sections were incubated for 24 h in GAM antiserum (Sigma-Aldrich GmbH) diluted 1:200 in the blocking solution. After rinsing, incubation for 24 h in peroxidase–antiperoxidase (PAP, diluted 1:40 in Tris buffer) followed. After thorough rinsing for several hours, peroxidase activity was developed with the 3,3′-diaminobenzidin (DAB; Sigma-Aldrich) enzyme reaction under microscopic control. For this purpose, sections were transferred to Tris buffer with a pH of 7.6. To 10 ml of buffer, 10 mg of ammonium nickel sulfate (FLUKA Chemie GmbH) and 5 mg of DAB were added. The enzyme reaction was initiated at a final concentration of 0.01% H_2_O_2_ and stopped with buffer after immunoreactive structures became visible. After rinsing, sections were embedded in Durcupan ACM, as described above. Ultrathin sections were only slightly contrasted with uranyl acetate (Ude and Agricola 1995).

Electron microscopic results were documented on Kodak Electron Microscope 4489 plates 8.3 × 10.2 cm. Plates were digitized with an Epson Perfection 4990 Photo Scanner at 1200 dpi.

### Antibody specificity and determination of cross-reactivity

Some of the antisera used in this study were prepared and processed by us. As shown in Table 1, the sera were developed in parallel in mice and rabbits. Sera were purified by precipitating the IgG fraction with ammonium sulfate (50% saturation) at 4 °C following dialysis against PBS overnight (see Harlow and Lane 1988). Antisera were mixed 1:1 with glycerol in 0.1 M of Millonig’s phosphate buffer stored in a freezer. Using a series of dilutions ranging between 1:500 and 1:10,000, the optimal working range for each serum was determined on tissue sections of the cockroach *Periplaneta americana.* Antibody specificity controls were performed in the following fashion: (i) Absence of staining after substitution of the primary antibody by non-immune normal serum. (ii) Absence of staining after pre-absorption (40–400 µg/ml) of the antiserum with corresponding antigen conjugate overnight in the refrigerator under constant shaking. (iii) Identical staining patterns after using specific antisera/antibodies from different sources, including mouse or rat antibodies. (iv) Possible cross-reactivities of the antibodies used were determined using an indirect competitive enzyme-linked immunosorbent assay (ELISA). Octopamine, for example, inhibits the binding of the monoclonal octopamine antibody by 100%, adrenaline by 63%, tyramine by 21%, and noradrenaline by 8%. In contrast, dopamine, DOPA, and serotonin did not cause any inhibition (Dacks et al. 2005). Cross-reactivities between the neuropeptide antisera tested were not observed, with two exceptions. First, the antiserum against allatostatin A7 recognizes all 14 isoforms of this neuropeptide found in the American cockroach. Secondly, the well-documented cross-reactivities for antisera against the RF group of neuropeptides were confirmed with the ELISA, but always in clear gradations (Agricola 1997; see: https://www.jenabioscience.com).

## Results

Of the 17 different antisera tested, only those directed against allatostatin A, proctolin, short neuropeptide F (sNPF; head peptide), octopamine, and serotonin showed clear immune labeling. The neuropeptides had been previously identified biochemically in the antennal heart (Predel et al. 1999; Predel 2001), as had the biogenic amine octopamine (Pass et al. 1988b). What is new is the detection of serotonin immunoreactivity and the absence of leucomyosuppressin immunoreactivity in the antennal heart.

### Allatostatin A immunoreactivity

**Fig. 1:**
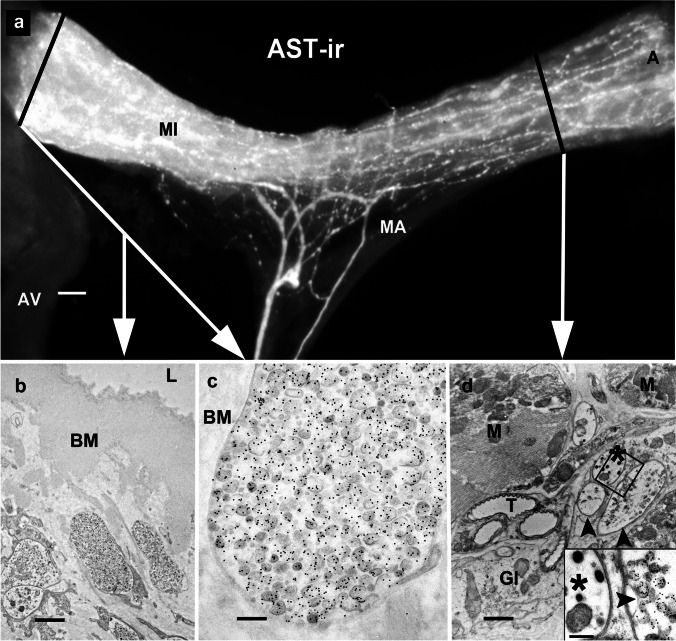
The antennal heart after light and electron microscopic immunostaining with a rabbit polyclonal antiserum against allatostatin-A. **a** Wholemount of the antennal heart showing immunoreactivity (AST-A-ir). Staining reveals axons, their ramifications, and varicose terminals on the *M. interampullaris* (MI) and close to the ampullae (A). The black lines approximately indicate the planes of sectioning for the electron micrographs shown in (**b)–(d**) (white arrows). Scale bar: 100 µm (**b**–**d**) Electron micrographs. **b** Section through the neurohemal tissue of the ampulla. All axon profiles and terminals show AST-A-ir. The immunoreactive profiles are embedded in a spongy connective tissue. A thick basement membrane (BM) separates the neurohemal tissue from the lumen of the ampulla (L). Scale bar: 1.7 µm. **c** With the protocol chosen, electron-light neurosecretory granules appear, often with membranes ruptured. Their mean diameter is approximately 120 nm. Scale bar: 250 nm** d** Electron micrograph from a transverse section through the. In addition to the AST-A-ir profiles with electron-light granules (arrowheads), unlabeled terminals with electron-dense granules are also visible (asterisk in the inset). The nerve fibers are enveloped by glial processes (Gl) and run between the muscle cells adjacent to tracheoles (T). Scale bar: 1.1 µm; inset: 250 nm

After immunolabeling, the paired antennal heart nerves stood out clearly from the *M. ampulloaorticus.* This paired muscle itself did not appear to be innervated by allatostatin A immunoreactivity (AST-A-ir) fibers. On the *M. interampullaris*, in contrast, a dense network of AST-A-ir nerve fibers appeared together with numerous terminals, especially in the area close to the ampullae (Fig. 1a).

Electron microscopic analysis showed numerous large secretion-filled terminals (2–6 µm) in the neurohemal tissue of the ampullae (Fig. 1b), close to the basement membrane. All axon terminals in the neurohemal tissue region of the ampulla showed granules labeled with gold grains. These round-to-oval AST-A-ir granules with a diameter of approximately 120 nm were relatively small for granules containing a neuropeptide. After fixation 2E, the neurosecretory granules were electron-light or vesicular, with the membrane often ruptured (Fig. 1c; Supplementary Fig. 3S). After fixation 1E, the neurosecretory granules appeared with an electron-dense core and a detached membrane (Fig. 6d). As expected from the light microscopic result (Fig. 1a), numerous AST-A-ir nerve profiles occurred in close association with the muscle fiber of the *M. interampullaris* (Fig. 1d).

### Proctolin-immunoreactivity

**Fig. 2:**
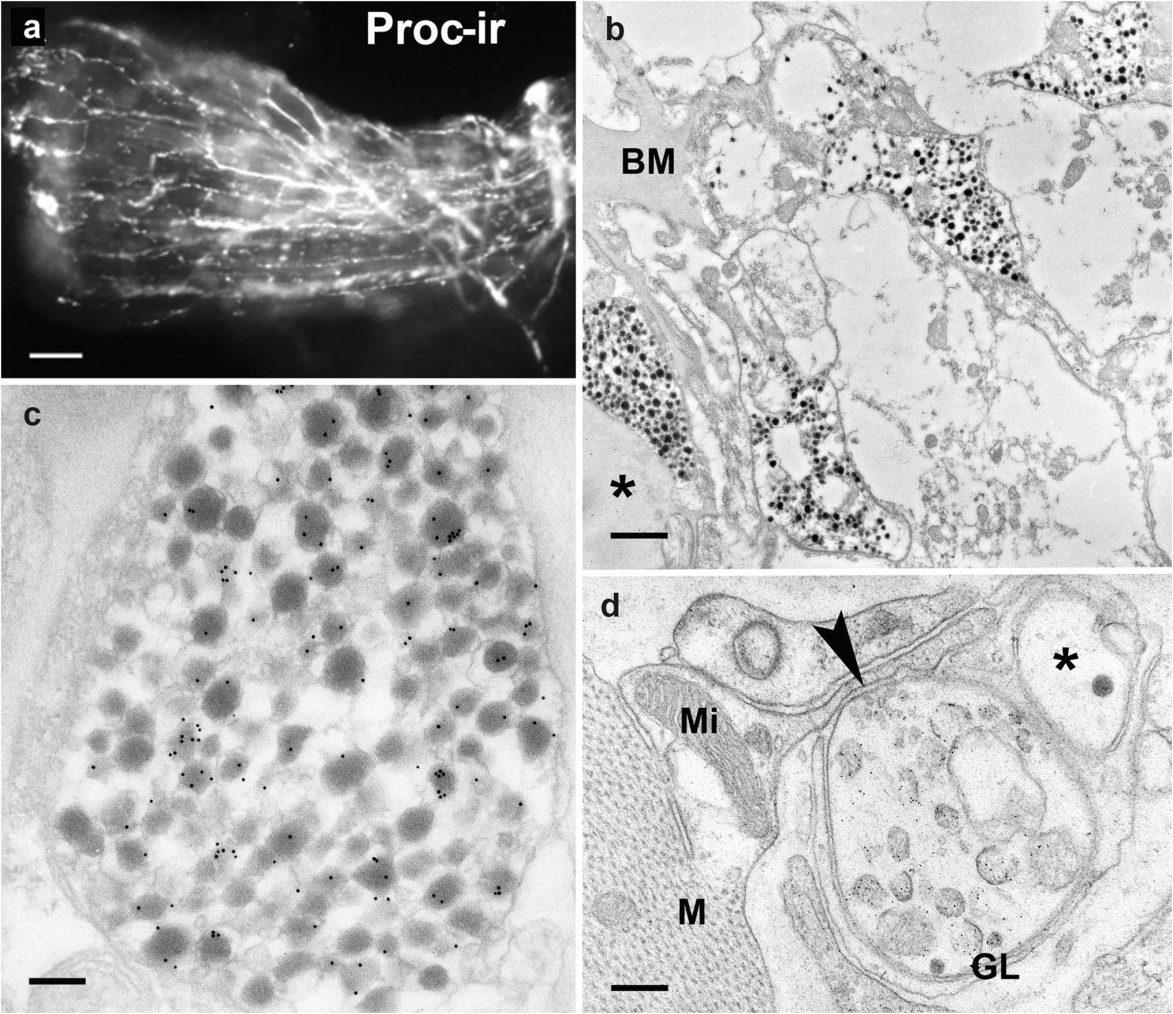
The antennal heart after light and electron microscopic immunostaining with a rabbit polyclonal antiserum against proctolin. **a** Fluorochrome labeling of PROC-ir nerve fibers of one-half of the *M. interampullaris* and one ampulla. Scale bar: 100 µm. **b** The neurohemal tissue of the ampulla. All axon profiles and terminals show PROC-ir. The PROC-ir fibers lie in a loose connective tissue. A few gold grains also appear on the basement membrane (BM; asterisk). Scale bar: 1 µm. **c** A PROC-ir terminal from the neurohemal area at higher magnification. Most of the neurosecretory granules are stained with gold particles. Scale bar: 150 nm. **d** Transverse section through the *M. interampullaris*. Close to the muscle (M), there is one labeled PROC-ir profile containing electron-lucent neurosecretory granules (see inset) and a second, unlabeled profile with one electron-dense granulum (asterisk). The arrowhead marks the release site of the PROC-ir fiber. Scale bar: 250 nm

Figure 2A shows the PROC-ir in one-half of the *M. interampullaris* and one ampulla. The axons of the antennal heart nerve branched on the muscle and extended to the wall of the ampulla. Here, they formed fluorescent dots, most likely neurosecretory boutons. In addition to the thick PROC-ir axons on the surface of the dilator muscle, a number of thin fibers occurred. For the first time, we successfully detected proctolin on the ultrastructural level using two different fixation variants. All axon terminals in the neurohemal tissue of the ampulla show a PROC-ir (Fig. 2b). Spherical or ellipsoid neurosecretory granules were about 120 nm in size, showed an electron-dense core, and had detached membranes. The axoplasm showed little or no immunolabeling (Fig. 2c). In the *M. interampullaris*, small PROC-ir profiles were found repeatedly, always associated with others showing no immunoreactivity (Fig. 2d).

### Serotonin immunoreactivity

**Fig. 3:**
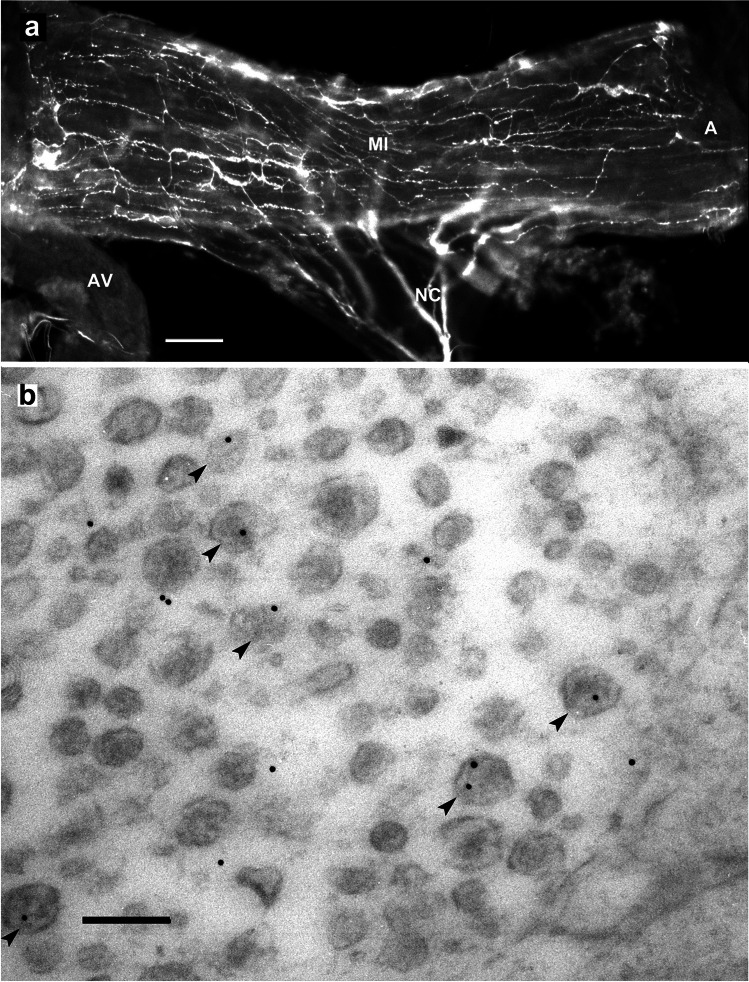
The antennal heart after light and electron microscopic immunostaining with a rabbit polyclonal antiserum to serotonin. **a** Fluorochrome labeling of 5-HT-ir nerve fibers of the *Nervus cardioantennalis* (NC) ramifying in numerous collaterals on both sides of the *M. interampullaris*. Scale bar: 100 µm. **b** Electron micrograph of a 5-HT-ir nerve fiber in the periphery of the antennal heart. Note six neurosecretory granules showing gold labeling (see arrowheads). Scale bar: 150 nm

Antisera to serotonin from mice and rabbits were used. Both yielded consistent results. The serotonin immunoreactivity (5-HT-ir) axons extended across the *M. interampullaris* to its insertion on the ampulla (Fig. 3a), forming many bouton-like terminals. Electron microscopic labeling reveals that some neurosecretory granules originating from the Golgi apparatus show gold grains, indicating 5-HT-ir (Fig. 3b, arrowhead). Most neurosecretory granules, however, are without immune labeling. All terminals in the ampullary neurohemal area showed 5-HT-ir.

### Octopamine immunoreactivity

**Fig. 4:**
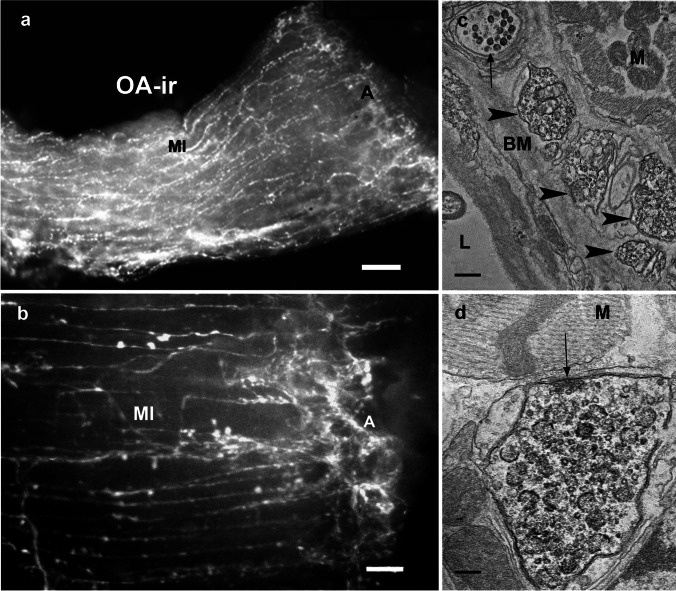
The antennal heart after light and electron microscopic immunostaining with a monoclonal octopamine antibody. **a** Fluorochrome labeling of OA-ir nerve fibers of one-half of the *M. interampullaris* and the ampulla. Note the longitudinal orientation of thin, beaded fibers, which run predominantly parallel to the dilator muscle fibers. Scale bar: 100 µm. **b** The regular appearance of varicosities or boutons along the entire length of the *Musculus interampullaris* (MI). Note the dense network of terminals arranged around the ampulla (A). Scale bar: 50 µm. **c** Electron microscopic imaging using the pre-embedding method. OA-ir profiles associated with the basement membrane (BM) of the muscle (M) are densely labeled with the peroxidase–antiperoxidase reaction product (arrowheads). No such profiles could be detected in the neurohemal region of the ampulla. In addition to the OA-ir fibers, unlabeled fibers with electron-dense granules are also seen in this region (arrow) Scale bar: 600 nm. **d** OA-ir nerve fibers also occur between the muscle cells of the *M. interampullaris*. Here, synaptic vesicles with a diameter of 45 nm characterize an exocytotic release site (arrow). Note the numerous clear vesicles in the OA-ir nerve fibers. A more strongly stained neurosecretory granulum is indicated (arrowhead). Scale bar: 250 nm

Numerous thin octopamine immunoreactivity (OA-ir) nerve fibers were found on the muscle fibers of the *M. interampullaris* (Fig. 4a) and likewise on those of the *M. ampulloaorticus*, where they merge with the *M. interampullaris* (Fig. 8b). The OA-ir fibers formed a particularly dense plexus at the ampulla (Fig. 4b). Larger axons were completely absent after OA-ir labeling. In comparison to AST-A-ir, PROC-ir, and 5-HT-ir labeling as described above, the OA-ir fibers appeared much thinner and emitted much weaker fluorescence that demanded long exposure times for detection. OA-ir fibers appeared to be absent from the neurohemal tissue of the ampulla. Electron microscopic immunocytochemistry was employed to clarify this point. While Stocker et al. (2018) succeeded with ultrastructural detection of OA-ir using the immunogold method, we were unsuccessful. We therefore employed the pre-embedding method to resolve the OA-ir structures. We found OA-ir nerve fibers of 0.5 to 1 µm in diameter (Fig. 4c) that occur in the periphery of the *M. interampullaris* together with unlabelled terminals of the same size (black arrow Fig. 4c). In contrast, our efforts to detect this amine in the neurohemal tissue of the ampulla were completely unsuccessful. A large number of small terminals filled with neurosecretory granules containing the PAP reaction product were always visible, close to but outside the ampulla. Additional OA-ir terminals were found on the muscle fibers of the *M. interampullaris*, some of them showing synaptoid structures (Fig. 4d).

### Short neuropeptide F immunoreactivity

**Fig. 5:**
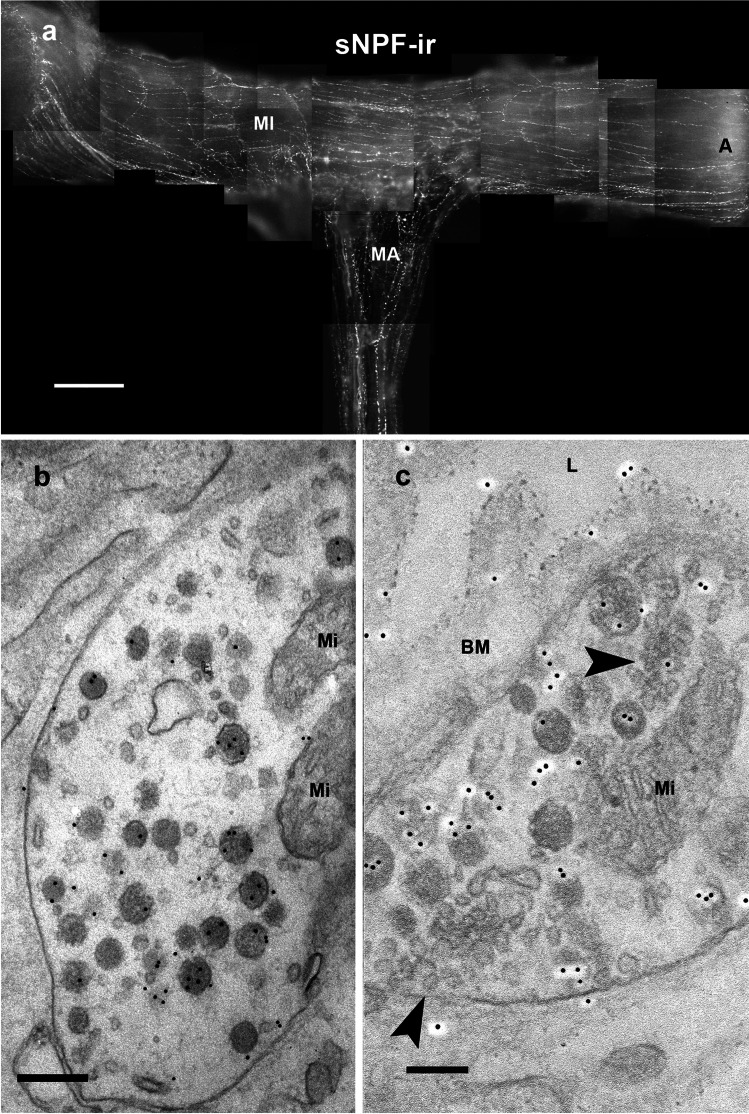
The antennal heart after light and electron microscopic immunostaining with a rabbit polyclonal anti-sNPF-antiserum. **a** Photomontage of 15 focal planes showing sNPF-ir on a wholemount of the antennal heart. A large number of thin, beaded sNPF-ir nerve fibers innervate both muscles of the antennal heart. Note that the fibers terminate near the rim of the ampulla and therefore do not reach the region with the neurohemal tissue. Scale bar: 100 µm. **b** Electron microscopic image of a sNPF ir-terminal in the peripheral region of the ampulla. Scale bar: 250 nm. **c** Peripheral sNPF-ir nerve fiber with two release sites (arrowhead) into the surrounding basal membrane and lumen of the head capsule (L). Note the immune labeling in the basal membrane (BM). See also sNPF-ir in the corpus cardiacum (Supplementary Fig. 4S) Scale bar: 150 nm

The immune response to the sNPF antiserum resembled the distribution of OA-ir. Many thin, short neuropeptide F-immunoreactive (sNPF-ir) nerve fibers innervated the paired *M. ampulloaorticus* bands along their entire length before they entered the *M. interampullaris*. On this muscle, they ran parallel to the muscle fibers toward the ampullae (Fig. 5a). At the ultrastructural level, sNPF-ir terminals located close to the rim of the ampullae were less than 1 µm in diameter and were filled with spherical, often vesicular, and weakly electron-dense granules of an average diameter of 110 nm (Fig. 5b, c; Supplementary Fig. 4S). Close to the basement membrane of the dilator muscle these terminals formed synaptoid structures and in such areas sNPF-ir often appeared in the basement membrane (Fig. 5c). No sNPF-ir fibers or terminals could be detected in the neurohemal tissue of the ampullae. To our knowledge, these are the first electron microscopic images of an sNPF-ir.

### Double labeling against allatostatin A and proctolin

**Fig. 6:**
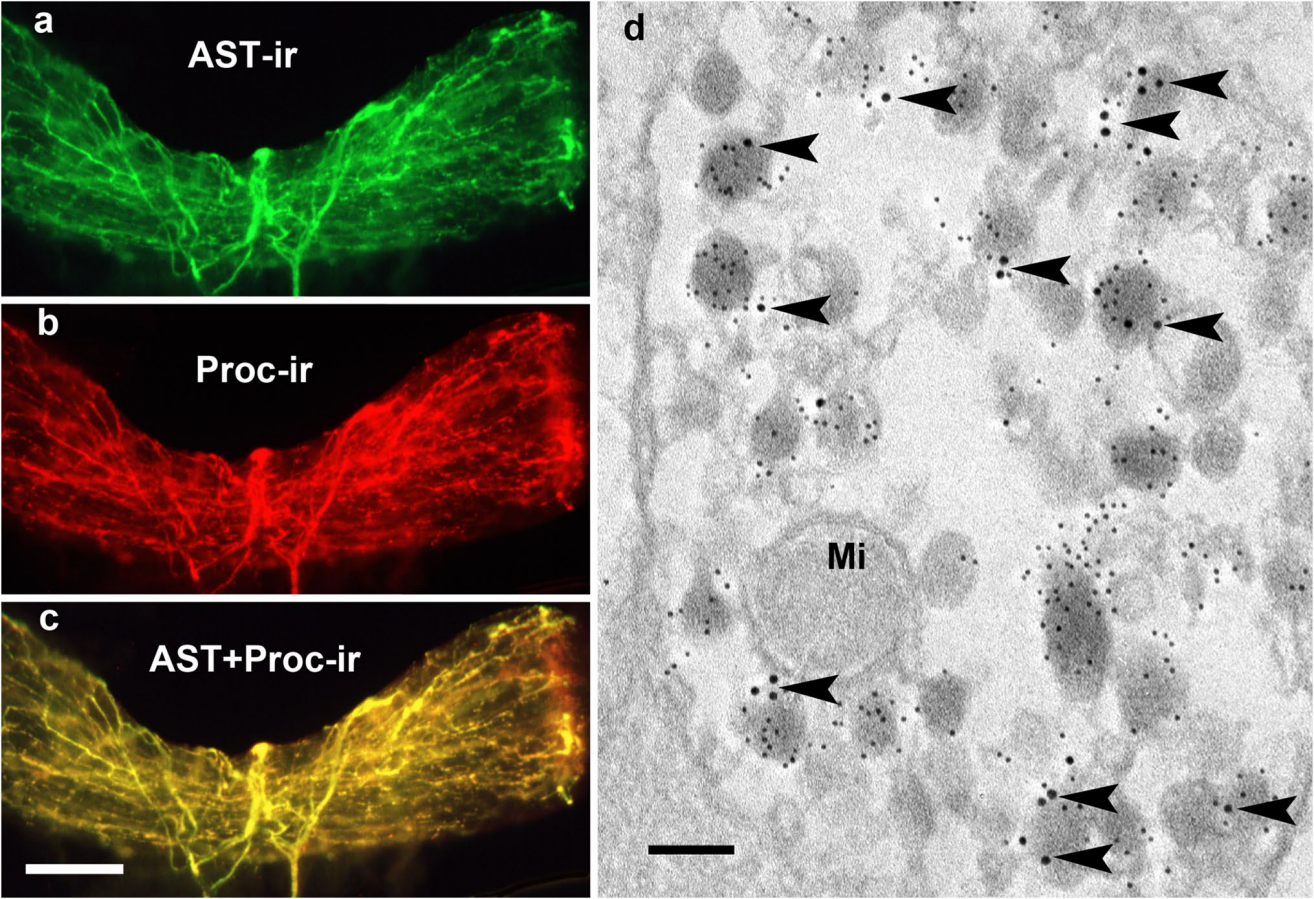
Co-localization of allatostatin-A and proctolin. **a** The antennal heart stained with a monoclonal AST-A antibody (green). The distribution of fluorescent fibers resembles the pattern shown in Fig. 1a. **b** The same preparation stained with a rabbit proctolin antiserum (red). The distribution of fluorescent fibers resembles the pattern shown in Fig. 2a. **c** Overlay of (**a**) and (**b**) shows that both antigens are present in the same nerve fibers (yellow). Scale bar: 100 µm. **d** Electron micrographs of AST-A-ir and PROC-ir in an axon terminal from the neurohemal area of the ampulla. Note both gold grain sizes of 6 nm (AST-A-ir) and 12 nm (PROC-ir) appear on the same neurosecretory granules (arrowheads). Scale bar: 90 nm

Wholemount preparations of the antennal heart after double staining showed co-localization of both neuropeptides in axons and their terminals in the ampulla region (Fig. 6a–c). Immunogold double staining revealed that in axon terminals in the area of the neurohemal tissue of the ampullae, almost half of all neurosecretory granules showed both gold grain sizes, indicating co-localization of both peptides in individual granules (Fig. 6d).

### Double labeling against allatostatin A and serotonin

**Fig. 7:**
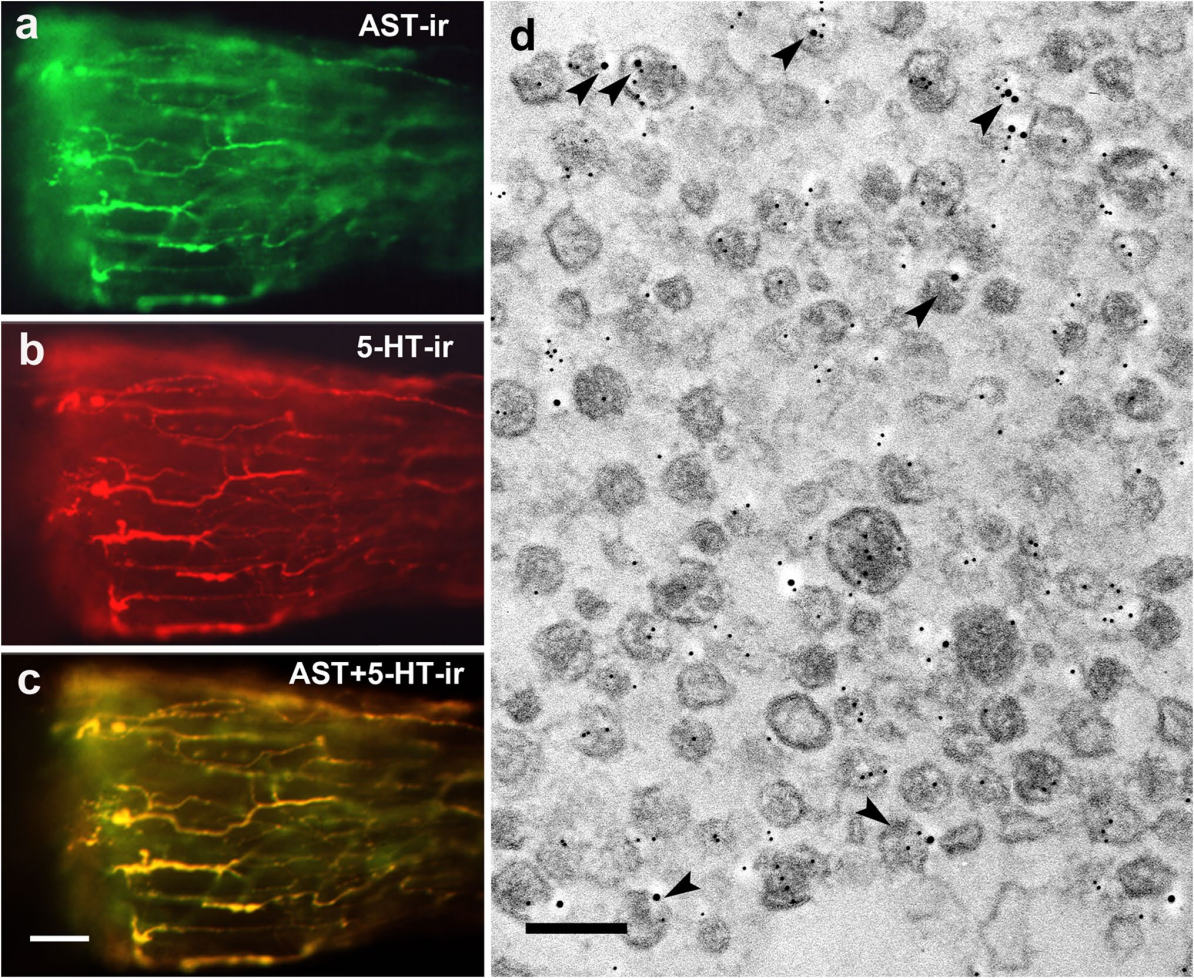
Co-localization of AST-A-ir and 5HT-ir. **a** One-half of an antennal heart stained with rabbit anti-AST-A antiserum (green). **b** The same preparation stained with a mouse anti-5-HT antiserum (red). **c** Overlay of (**a**) and (**b**) shows that both antigens are present in the same nerve fibers (yellow). Scale bar: 50 µm. **d** Electron micrograph of AST-A-ir (Au_6_) and 5-HT-ir (Au_12_). Six double-labeled granules are marked by arrowheads. Scale bar: 150 nm

The antennal heart after double staining showed a staining pattern very similar to the one observed after staining against AST-A and proctolin (Fig. 7a–c; compare with Fig. 6a–c). In Fig. 7a–c, the left side of a wholemount specimen of the antennal heart is shown. In Fig. 7a, it is stained with a mouse AST-A antibody (green), and in Fig. 7b, it is stained with a rabbit serotonin antiserum. In Fig. 7c, the two immune reactions are exposed one after the other (merged). Due to their yellow color, the axons show co-localization of AST-A-ir and 5-HT-ir. In some specimens with this double staining, there are deviations in the two staining patterns, but always within identical axons. At the ultrastructural level, both antigens were detectable by gold grains of different sizes (Fig. 7d). A large proportion of the neurosecretory granules showed AST-A-ir, but a few of them also showed 5-HT-ir. This indicated the co-localization of AST-A and 5-HT in individual neurosecretory granules (Fig. 7d, arrowheads). Serotonin labeling also appeared in the axoplasm.

### Double labeling against allatostatin A and octopamine

**Fig. 8:**
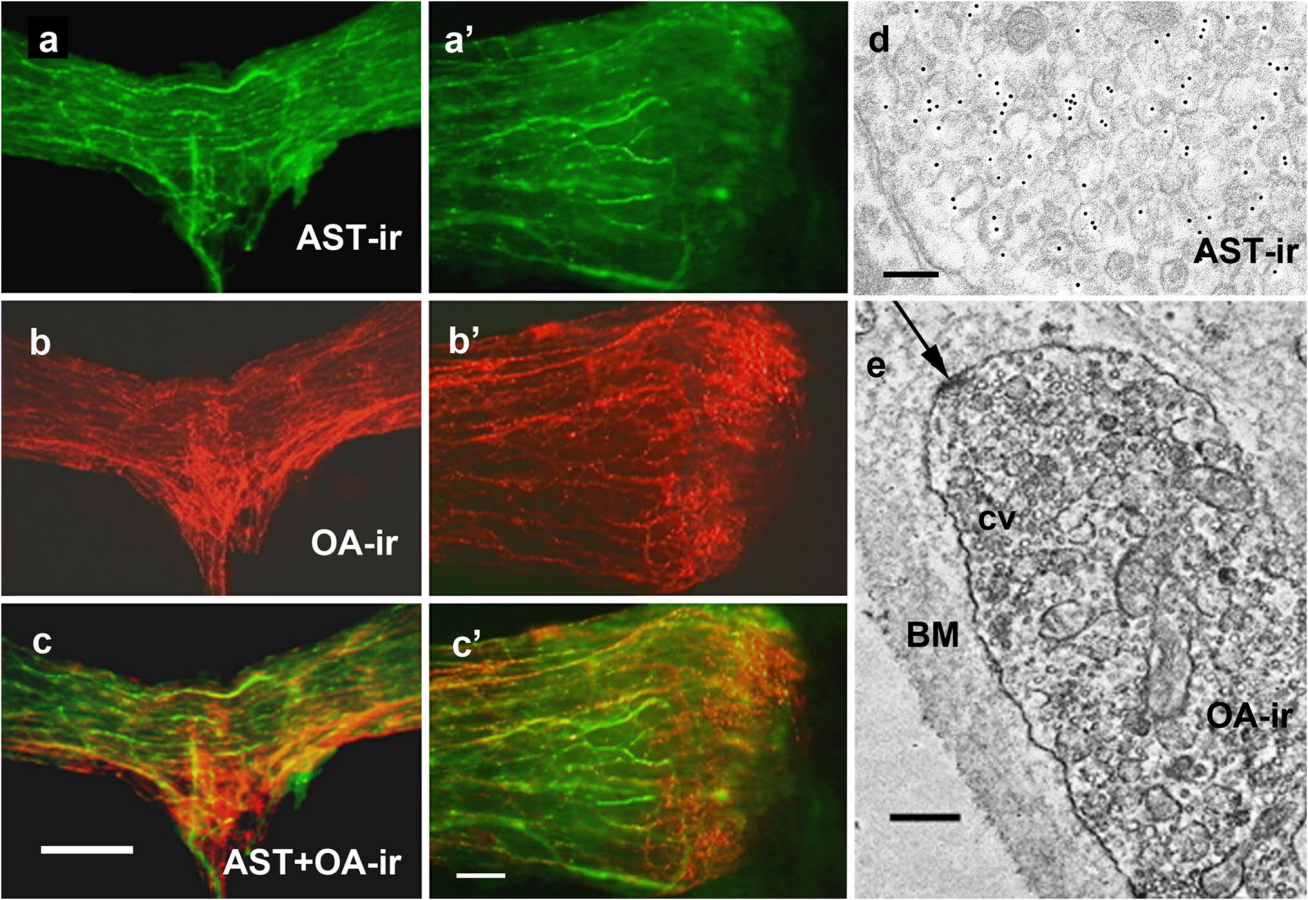
OA and AST-A are not co-localized. Light and electron microscopic immunostaining with a monoclonal octopamine antibody and rabbit anti-allatostatin-A serum. **a** Middle part of the antennal heart after visualization of the AST-A-ir. **b** The same after visualization of OA-ir (red, compare with Fig. 4). **c** The overlay image shows that the AST-A-ir and the OA-ir appear in different fiber systems. Scale bar: 100 µm. **a’–c’** Even at higher magnification of the area of the ampulla, no co-localization of AST-A-ir with OA-ir can be seen. Scale bar: 50 µm. **d**, **e** Electron microscopic double immunostaining by coupling the pre-embedding PAP technique with the post-embedding immunogold method. Shown are two different parts of the same ultrathin section. AST-A-ir visualized with immunogold (AU_10_) octopamine visualized by the pre-embedding PAP technique. The AST-A-ir fibers in (**d**) in no case show the PAP reaction product. Vice versa, the OA-ir-fibers shown in (**e**) do not show any gold labeling. **d** Terminals in the area of the neurohemal tissue of the ampulla. Scale bar: 400 nm (× 20,000).** e** OA-ir nerve fibers can only be visualized in the peripheral area of the *M. interampullaris* but not in the neurohemal tissue of the ampulla. (Note the numerous clear vesicles (cv) and synaptoid structures (arrow), sites of the release of OA into the hemolymph of the head capsule through the basement membrane (BM)). Scale bar: 150 nm

As described above, the overall staining pattern of OA-ir structures differed markedly from that of allatostatin A, proctolin, and serotonin. The observation that AST-A-ir and OA-ir occurred in separate nerve fiber systems (Fig. 8a–c) supported this difference. There was no overlap between the two systems, so allatostatin A and octopamine were not co-localized. Electron microscopy confirmed this finding. Post-embedding immunogold labeling for allatostatin A (Fig. 8d) was possible even after the procedures of pre-embedding immunolabeling for octopamine (Fig. 8e). Thus, on the ultrastructural level also, double staining of identical terminals was not found in any case.

### Double labeling against allatostatin A and short neuropeptide F

**Fig. 9:**
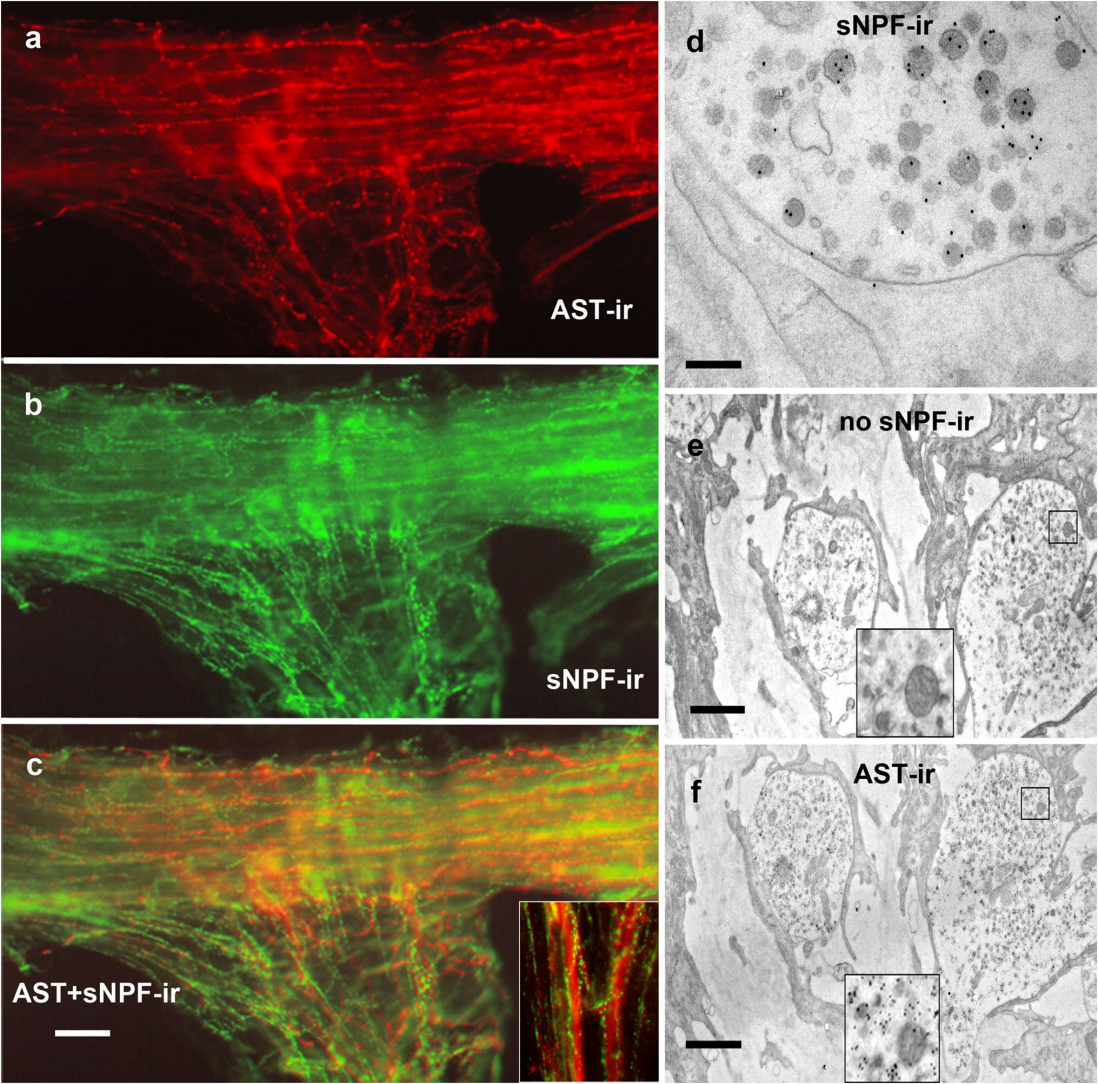
AST-A and sNPF are not co-localized. **a** Middle part of the antennal heart after visualization of the AST-A-ir (red). **b** The same after visualization of anti-sNPF-serum (green). **c** The overlay image shows that the AST-A-ir and the sNPF-ir appear in different fiber systems. Scale bar: 100 µm. **d** Ultra-thin section with immunogold staining of a sNPF-ir profile in the periphery of the *M. interampullaris* (AU_12_). Scale bar: 250 nm. **e** First of two consecutive ultra-thin sections. No sNPF-ir is visible in the axon terminals of the ampulla neurohemal tissue. **f** On the following ultrathin section, AST-A-ir is clearly visible in the axon terminals (AU_12_). Scale bar: 1.7 µm. The insets provide views of a small area at higher magnification

AST-A-ir and sNPF-ir were also not co-localized in the *M. interampullaris* (Fig. 9). The thin sNPF-ir nerve fibers accompanied and surrounded the thicker AST-A-ir nerve fibers (Fig. 9c, inset). To exclude co-localization in the neurohemal tissue of the ampulla, we employed electron microscopy. We stained one ultrathin section of this tissue with the AST-A antibody and the following one with the anti-sNPF serum. The results clearly showed that no co-localization of these two neuropeptides occurred within the terminals of the neurohemal tissue (Fig. 9e, f).

### Double labeling against octopamine and short neuropeptide F

**Fig. 10:**
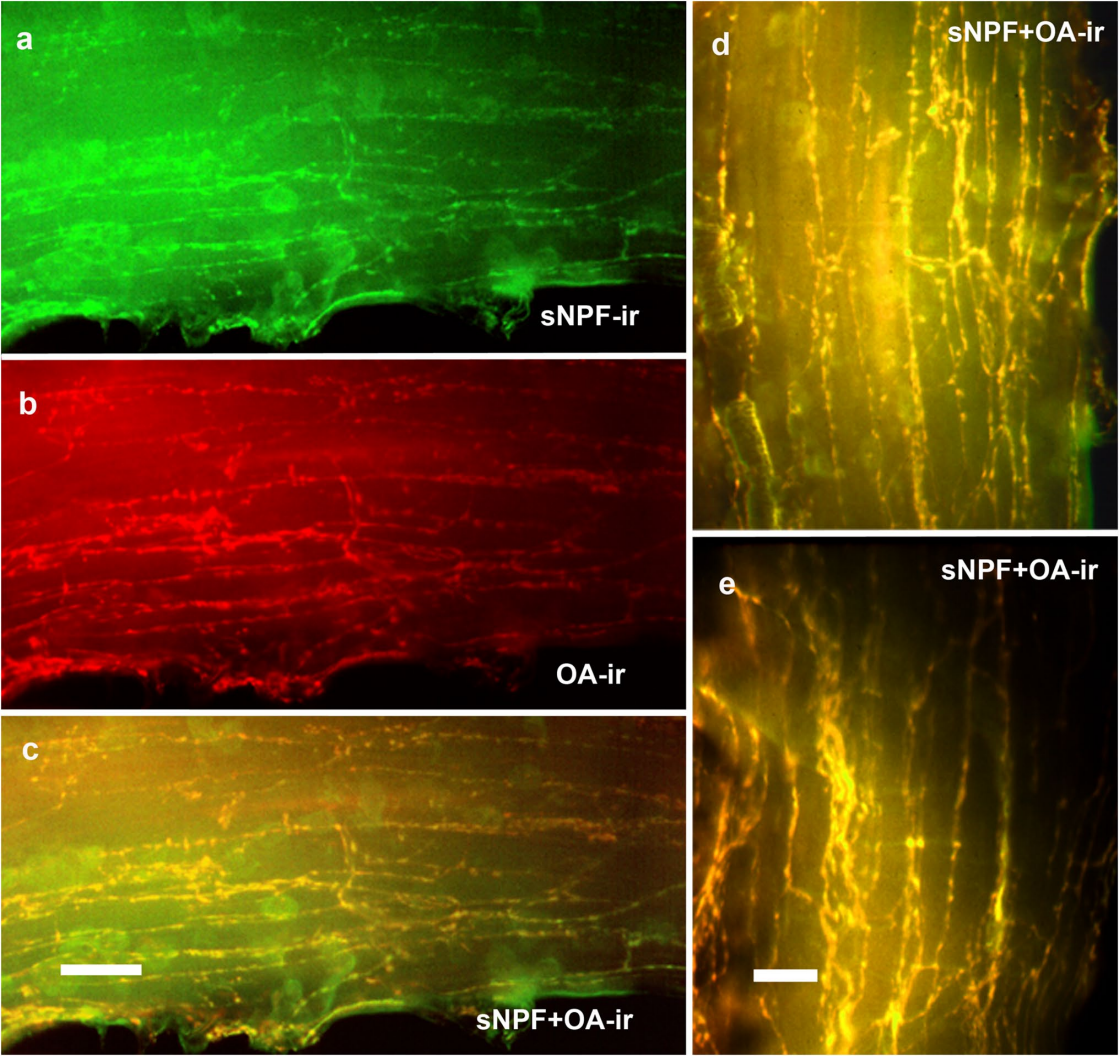
Co-localization of sNPF and OA. **a** Middle part of an antennal heart stained with rabbit anti-sNPF serum (green). **b** The same preparation stained with a monoclonal antibody against octopamine (red). **c** Overlay shows that both antigens are present in the same nerve fibers (yellow). Scale bar: 50 µm. **d**, **e** Different focal planes at higher magnification. Scale bar: 25 µm

Double labeling showed a clear overlap of the sNPF-ir with OA-ir (Fig. 10). Thus, sNPF appeared to be co-localized with the OA. In both cases, the fibers were thin and emitted weak fluorescent signals. This was in clear contrast to the fiber system labeled with antisera against allatostatin A, proctolin, and serotonin, as described above.

### Electron microscopic immunostaining of sNPF, perisulfakinin, leucomyosuppressin), and AST-A in the corpora cardiaca

**Fig. 11:**
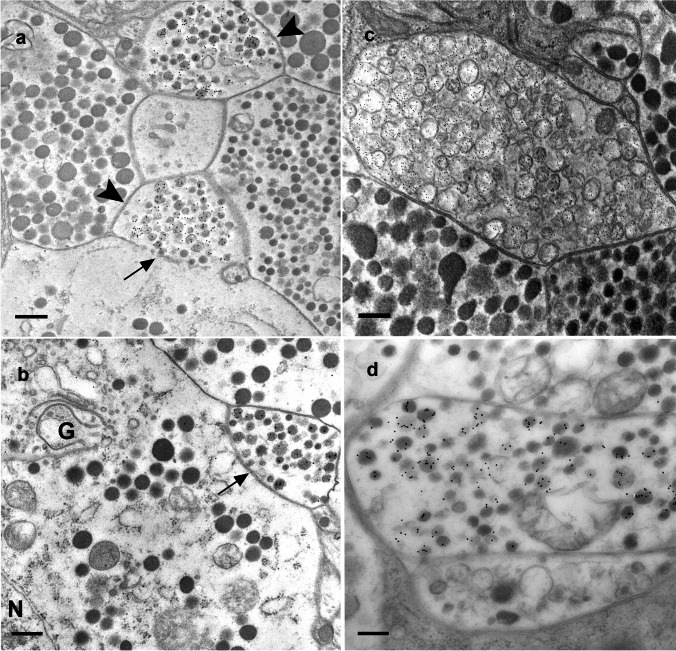
Electron micrographs of sNPF-ir (**a**), AST-A-ir (**b**), perisulfakinin-ir (**c**), and leucomyosuppressin-ir (**d**) in the corpus cardiacum. The perisulfakinin-ir (AU_6_) and the leucomyosuppressin-ir (AU_12_) are predominantly present in axon terminals of the storage lobe. AST-ir (AU_12_) is detectable throughout the corpus cardiacum, while the sNPF-ir seems to be restricted to the glandular part of the corpus cardiacum. The allatostatin-A and sNPF-ir (AU_12_, Supplementary Fig. 4S) nerve fibers often border directly on the glandular cells (arrows) or on their extensions (arrowhead). Scale bars: **a** 400 nm, **c** 250 nm, **b** 330 nm, and **d** 220 nm

On their way to the antennal heart, the axons of the neurons in the suboesophageal ganglion form collaterals within the corpora cardiaca (Pass et al. 1988a). For this reason, neuropeptides that were detected in the antennal heart should also be detectable in the corpora cardiaca. Accordingly, sNPF-ir axon terminals could be detected predominantly in the glandular part of the corpora cardiaca (Fig. 11a). There is direct contact with the glandular cells or their extensions. Often, sNPF-ir also appeared in the surrounding basement membrane (Supplementary Fig. 4S). Leucomyosuppressin (LMS)-ir, which we could not detect in the antennal heart, was detected in the axon terminals of the corpora cardiaca, predominantly in the storage lobe (Fig. 11d). However, the basement membrane did not appear marked in any case. The perisulfakinin (PSK) antiserum, as another one out of the group of antisera against RFamide-like peptides, recognized all known RF-amides, but with a clear gradation. PSK-ir appears in numerous axon terminals of the corpora cardiaca but exclusively in the storage lobe (Fig. 11c). Due to their electron-lucent matrix, the PSK-ir neurosecretory granules differed significantly from the other RF-amide-positive ones described above. The immunolabels for LMS, PSK, and sNPF are specific (see antibody specificity). AST-A-ir could be detected in all parts of the corpora cardiaca. Direct contact of an AST-A-ir axon terminal with a glandular cell is shown in Fig. 11b.

## Discussion

### The antennal heart neurons and their neuromediators

**Fig. 12:**
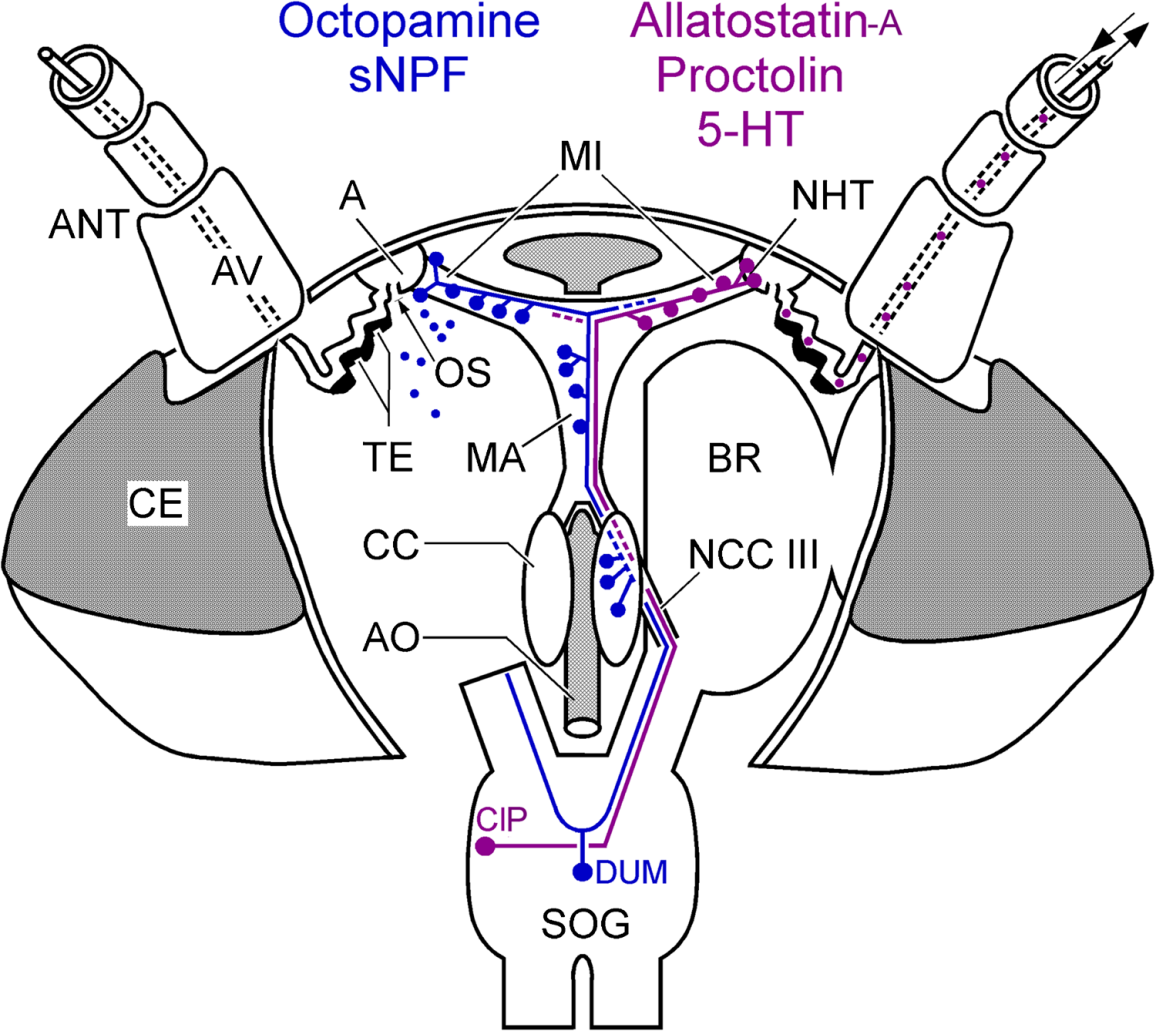
Schematic representation of the antennal heart and its innervation (based on Pass 1998). The *Musculus interampullaris* (MI) is suspended between the ampullae (A) and dilates these when contracting. The *Musculus ampulloarticus* (MA) connects the ampullae to the aorta (AO). Each ampulla is connected to the lumen of the head by an ostium (OS). Originating from each ampulla, an antennal vessel (AV) runs into the antenna (ANT). The wall of the vessel is thickened in its proximal region and looks like a transport epithelium (TE). The antennal heart is innervated by two types of neurons, with somata located in the suboesophageal ganglion (SOG). There is one set of two bilaterally paired neurons with somata contralateral to their axons (magenta). Second, there are one or two unpaired median neurons with bifurcating axons (blue). For reasons of clarity, only one neuron is shown for each type. The axons enter the connective toward the brain (BR) and continue through one branch of the nervus corporis cardiaci III. This branch passes through the corpus cardiacum (CC) and along the aorta to reach the two muscles and the ampullae. Each one of the neurons again bifurcates in the periphery to innervate both halves of the *M. interampullaris*. Only one branch is shown. Where the *M. interampullaris* connects to the ampullae, neurohemal tissue is formed (NHT), opening into the lumina of the ampullae. CA, corpus allatum; CE, compound eye

Our detailed analysis of the neuromediators innervating the antennal heart of the cockroach revealed two systems. In the first, octopamine is co-localized with sNPF, and in the second, serotonin is co-localized with A-type allatostatins and proctolin (Fig. 12). The most plausible source for octopamine is the two dorsal unpaired median (DUM) neurons in the suboesophageal ganglion (SOG) shown to innervate the antennal heart (Pass et al. 1988a). All large DUM neurons in this ganglion have been shown to be octopaminergic in cockroaches and other insects (Stevenson and Spörhase-Eichmann 1995; Bräunig and Pflüger 2001). It follows from this that the somata of at least two of the DUM neurons in the SOG should also contain sNPF, although this remains to be investigated. The contralateral paired (ClP) neurons in the SOG revealed after retrograde staining of the antennal heart nerve (Pass et al. 1988a) must be the source of serotonin, allatostatin, and proctolin. This notion is further supported by the fact that immunocytochemical staining of vibratome sections of the SOG revealed a pair of somata in the anterior ventral region that showed both AST-A-ir and PROC-ir (Supplementary Fig. S1). These somata, by their size and position, matched those of the ClP somata described previously. Even more important, they were the only somata in the entire ganglion that contained both mediators, just like the one set of fibers and terminals observed in the antennal heart.

The co-localizations observed by light microscopy were corroborated by our ultrastructural investigations. Moreover, these showed that different mediators may not only be co-localized in one neuron but also in the same neurosecretory granules. The pre-embedding method showed OA-ir terminals of an average size of 1 μm containing granules of an average diameter of 110 nm (Figs. 4d and 8e). Granules of 110 nm also exhibited sNPF-ir after the post-embedding immunogold labeling (Fig. 5a, c). Because of the two different staining methods, double labeling at the ultrastructural level did not appear feasible. Taken together, our light and electron microscopic results indicate that octopamine and sNPF are co-released from the same granula.

Terminals displaying AST-A-ir are up to 5 µm in size and contain granules with an average diameter of 120 nm (Figs. 1b–d and 7d). The same applies to granules showing PROC-ir (Fig. 2b–d). Double labeling showed that both AST-A-ir and PROC-ir occurred in the same granule (Fig. 6d). Surprisingly, we also observed serotonin and allatostatin in individual granules (Fig. 7d). Haller (1992) reported a co-localization of serotonin and a neuropeptide, the calcitonin gene-related peptide, in individual neurosecretory granules. Haller’s (1992) and our observation illustrate what appears to be a rare phenomenon. Peptides find their way into granules by way of the Golgi apparatus and the secretory pathway; biosynthesis of biogenic amines, in contrast, takes place in the cytoplasm. Co-localization can only be achieved by an uptake mechanism that transports serotonin into the peptide-containing granules. Our results do not allow for any estimation of the ratios in which these three neuromediators are contained in the granules or whether these ratios remain constant over time. It was shown recently that the neuromediator content in granules may vary (Massah et al. 2022). With its two different systems of co-localized neuromediators, the antennal heart appears to be an attractive system for future studies addressing such possible variations in transmitter levels and their causes.

### The antennal heart neurons and their targets

Figure 12 summarizes our findings with respect to the innervation of substructures of the antennal heart. The DUM neurons innervate the *M. interampullaris* and the two strands of the *M. ampulloaorticus*. Their terminal ramifications are particularly dense at the outer margins of the ampullae, at the insertions of the *M. interampullaris*. They do not innervate the neurohemal tissue on the inside of the ampullae. The ClP neurons innervate the *M. interampullaris* only and in addition terminate in the neurohemal tissue of the ampullae. From here, their neuromediators could be transported directly into the lumen of the antennal vessel and, in this way, distributed within the whole antenna. It was shown previously that both types of neurons also form terminal ramifications in the corpora cardiaca (Pass et al. 1988a).

Apart from the direct route into the antennal vessel from the neurohemal tissues within the ampullae, an indirect route is also possible. Neuromediators might spill over from the muscles and enter the ampullae through their ostia. This might be important for octopamine and sNPF, which might find their way into the nearby ostia from the particularly dense ramifications of the DUM neurons close to the ampullae. Our finding of sNPF-ir in the basement membrane (Fig. 5c; Supplementary Fig. 4S) supports this notion. A further target of all mediators could be the specialized transport epithelium that was found in the proximal region of the antennal vessel (Pass 1985, Pass et al. 1988a). A schematic representation of the pathways of all five neuromediators is provided in Supplementary Fig. 2S.

### Possible actions of the neuroactive substances on antennal heart structures

Previously observed effects of octopamine, proctolin, and allatostatin on the antennal heart muscles have been listed in the **Introduction**. Surprisingly, we could not find any fibers immunoreactive to LMS because this peptide had been shown to strongly affect the antennal heart (Hertel 1994). With biochemical methods, this peptide was detected in some, but not all, preparations of the antennal heart muscles (Predel 2001). We detected numerous LMS-ir axon profiles in the storage lobe of the corpus cardiacum (Fig. 11c). Such fibers might extend along the aorta and reach antennal heart muscles in some but not all specimens, which might explain this variability. The effects of sNPF on the antennal heart muscles need to be investigated.

### Possible actions on antennal structures

As already mentioned, the three mediators contained in the ClP neurons, serotonin, allatostatin, and proctolin, could enter the antennal vessel and this way the entire antenna via the neurohemal tissue in the ampullae of the antennal heart. Octopamine and sNPF could perhaps also enter the antennae by the indirect route described above (Fig. 12). So far, there exists no direct evidence for either proctolin or allatostatins A to exert direct influences on the antennal sensilla. However, in the cockroach *Blatella germanica*, injection of allatostatin A resulted in a drastic reduction in food consumption (Aguilar et al. 2003). In *Drosophila melanogaster*, the activity of allatostatin A-containing neurons (or neuroendocrine cells) promotes food aversion and inhibits feeding behavior (Hergarden et al. 2012). Possible direct modulation of food odor-sensitive sensilla by allatostatin A peptides released into the antennal vessel would make sense in the context of feeding and satiety (Wegener and Chen 2022).

Serotonin also is an important player in the control of feeding and satiety in both vertebrates and invertebrates (for review, see Tecott 2007; Vleugels et al. 2015; Bacqué-Cazenave et al. 2020; Tierney 2020; van Galen et al. 2021). Our finding that both the antennal heart muscles as well as its neurohemal tissue are innervated by serotonin-immunoreactive fibers is new, and it fits well into this context. Both cockroaches and locusts possess an extended serotonergic neurohemal system (Bräunig 1987; Davis 1987) that covers the surface of all peripheral nerves of the head. In locusts, the neurons forming this network have been shown to be active during feeding (Schachtner and Bräunig 1993). In several insect species, feeding caused an increase in hemolymph serotonin levels, and injection of serotonin caused a reduction in food consumption (Tierney 2020). Elevated levels of serotonin in the hemolymph of the head together with serotonin released directly into the antennal vessel by the ClP neurons could raise the level within the antennae. Evidence that serotonin modulates olfactory sensory cells is sparse (Küppers and Thurm 1975; Grosmaitre et al. 2001; Dolzer et al. 2001; Siju et al. 2008; Watanabe et al. 2014), but receptors for serotonin within the antennae have been documented in several insect species (Pitts et al. 2011; Mohapatra and Menuz 2019; Latorre-Estivalis et al. 2020). Influences of serotonin on food-odor-specific olfactory receptors should be the topic of future investigations.

As described above, octopamine and sNPF may enter the lumen of the antennae via an indirect route. Our ultrastructural investigation corroborates the finding that there is a high concentration of octopamine around the ampullae but does not support its presence in the ampullary neurohemal tissue (Pass et al. 1988b). Octopamine also is a main player in the control of insect energy metabolism (reviewed by Agricola et al. 1988; Farooqui 2007; Roeder 2020). Direct and indirect effects of octopamine on olfactory receptor neurons have been described in many cases (Pophof 2000; Grosmaitre et al. 2001; Zhukovskaya and Kapitsky 2006; Flecke and Stengl 2009; Zhukovskaya 2012; Jung et al. 2013; Zhukovskaya and Polyanovsky 2017), and octopamine receptors expressed in antennal cells (receptor cells and others) were found in several insect species (Brigaud et al. 2009; Latorre-Estivalis et al. 2020; Finetti et al. 2023). In *Periplaneta*, olfactory sensitivity shows daily fluctuations mediated by octopamine and tachykinin (Jung et al. 2013). Interestingly, in *Bombyx mori*, octopamine enhanced responses in sensory neurons tuned to sex pheromone components, but not in those for general odorants (Pophof 2002). This illustrates that neuromodulatory effects cannot be generalized to the entire populations of receptor neurons and that the plasticity of the peripheral olfactory system of insects is a matter that is most complicated (for review, see Sengupta 2013; Gadenne et al. 2016).

In 1995, Veenstra and Lambrou isolated and sequenced the “head peptide” from Periplaneta (ANRSPSLRLRFa) (Veenstra 2014). The antibody used in the work was developed against this peptide. The high sequence similarity of the head peptide with the *Drosophila* sNPF (ASRSPSLRLRFa, Fadda, et al. 2019) shows that two names exist for the same neuropeptide. We have chosen the name sNPF. The short neuropeptide F also influences many aspects of feeding, satiety, and energy metabolism, and the direct modulation of olfactory receptor neurons by this peptide is documented at least for flies (Nässel and Wegener 2011; Schoofs et al. 2017; Jiang et al. 2017; Nässel and Zandawala 2019; Fadda et al. 2019; Liu et al. 2021). For other insects, the overall picture is less clear (Root et al. 2011; Dillen et al. 2014; Bestea et al. 2022; Liu et al. 2021; Amir et al. 2022).

### ClP and DUM neuron collaterals in the retrocerebral complex

Retrograde staining of the antennal heart nerve (Pass et al. 1988a) did not only reveal the neurons in the subesophageal ganglion but also numerous fine arborizations within the corpus cardiacum. The main neurohemal organ is thus an additional release site for the neuromediators produced by the two sets of neurons innervating the antennal heart. Our ultrastructural investigation supports this notion, as it showed that sNPF-ir and AST-A-ir nerve fibers directly innervate the glandular cells of the corpus cardiacum (Fig. 11a, b). In the locust *Locusta migratoria*, allatostatin A acts in a dose-dependent manner as a releasing factor for the adipokinetic hormone AKH (Clark et al. 2008), the key hormone for the regulation of energy metabolism in insects. Also in locusts, a similar effect was described for proctolin (Clark et al. 2006). Although direct evidence is lacking, the ClP neurons, releasing both allatostatin A and proctolin, may also be involved in the release of AKH in the American cockroach. An opposite effect is described for the sNPF. In *Drosophila*, the paired sNPF-containing CN neurons project to the corpus cardiacum and inhibit AKH release (Oh et al. 2019). If sNPF is generally involved in the inhibition of AKH secretion, this could also be one of the functions of the sNPF-ir DUM neurons described here. Intriguingly, however, octopamine, the second transmitter of the DUM neurons, triggers the secretion of the AKH in locusts (Pannabecker and Orchard 1986). Thus, the molecular mechanisms of the combinatorial effects of octopamine and sNPF need further elucidation.

## Supplementary Information

Below is the link to the electronic supplementary material.

Supplemental Table 1S (DOCX 10 kb)

Supplementary Fig. 1S Co-localization of allatostatin and proctolin in a single neuronal soma within the suboesophageal ganglion (SOG) **A** Parasagittal vibratome section of the SOG marked with AST A-ir (red). **B** The same section stained with PROC-ir (green). **C** Overlay of **A** and **B** shows that both antigens are present in a single soma (yellow). This soma is the only one within the SOG carrying both labels and corresponds in size and position the ClP soma described previously (Pass et al. 1988a). (PNG 3419 kb)

High resolution image (TIF 29897 kb)

Supplementary Fig. 2S Schematic representation of the pathways by which the neuromediators contained in the DUM and ClP neurons could reach substructures of the antennal heart and other targets. (PNG 226 kb)

High resolution image (TIF 11870 kb)

Supplementary Fig. 3S Electron micrograph of an AST A-ir terminal of the ampulla. At high magnification and weak osmification (0.05% OsO_4_), the matrix of the neurosecretory granules appears vesicular. Scale bar: 155 nm (PNG 17493 kb)

High resolution image (TIF 2756 kb)

Supplementary Fig. 4S Electron micrograph of an sNPF-ir terminal in the corpora cardiaca. Note the immu-noreactivity in the area of the basal lamina. Scale bar: 250 nm (PNG 1811 kb)

High resolution image (TIF 16845 kb)

## Abbreviations

5-HT: 5-Hydroxytryptamine = serotonin
A: Ampulla
AST-A: Allatostatin A 1–14
AV: Antennal vessel
Au: Gold
BM: Basement membrane
BSA: Bovine serum albumin
CCAP: Crustacean cardioactive peptide
ClP: Contralateral paired (-neuron)
CY2, CY3: Fluorescent carbocyanine tags
ddH_2_O: Double-distilled H_2_O
GABA: *gamma*-Aminobutyric acid
GAM: Goat antimouse
DAB: Diaminobenzidine
DUM: Dorsal unpaired median (-neuron)
GAR: Goat antirabbit
GL: Glia
ir: Immunoreactive, immunoreactivity
L: Lumen
*M. ampulloaorticus*: *Musculus ampulloaorticus*
*M. interampullaris*: *Musculus interampullaris*
Mi: mitochondrion
NC: *Nervus cardioantennalis*
NGS: normal goat serum
sNPF: short neuropeptide F
OA: octopamine
PAP: peroxidase–antiperoxidase
PBS: phosphate-buffered saline
PROC: proctolin
SMB: sodium metabisulfite
T: trachea, tracheae
TB: Tris buffer
Tx: Triton X-100
