# Supplementary material for: The complex neurochemistry of the cockroach antennal heart
Source: Cell Tissue Res. 2024 Sep 6;398(2):139–60. doi: 10.1007/s00441-024-03915-5 (PMC11525290; doi:10.1007/s00441-024-03915-5)
Supplement: Supplementary file 1 — (DOCX 10 kb) [file 441_2024_3915_MOESM1_ESM.docx]

**Supplementary Table 1**

| **Protocol variants of tissue treatment by image number** | | | | |
| --- | --- | --- | --- | --- |
| **Figure** | **protocol** | **addition 1** | **addition 2** | **visualization** |
| 1A | 2L |  |  | GAR-Cy3 (AST-A) |
| 1B | 2E |  | 0.05% OsO_4_ | GAR Au_12_ (AST-A) |
| 1C | 2E |  | 0.05% OsO_4_ | GAR Au_12_ (AST-A) |
| 1D | 2E |  | 0.05% OsO_4_ | GAR Au_12_ (AST-A) |
| 2A | 1L |  | 1% OsO_4_ | GAR-Cy3 (Proc) |
| 2B | 1E |  | 1% OsO_4_ | GAR Au_10_ (Proc) |
| 2C | 1E |  | 1% OsO_4_ | GAR Au_10_ (Proc) |
| 2D | 1E | NaBH_4_ | 1% OsO_4_ | GAR Au_10_ (Proc) |
| 3A | 2L |  |  | GAR-Cy3 (5-HT) |
| 3B | 2E |  | 0.05% OsO_4_ | GAR AU_12_-(5-HT) |
| 4A | 1L |  |  | GAM-Cy3 (OA) |
| 4B | 1L |  |  | GAM-Cy3 (OA) |
| 4C | 3E |  | 0.5% OsO_4_ | PAP-DAB (OA) |
| 4D | 3E |  | 0.5% OsO_4_ | PAP-DAB (OA) |
| 5A | 2L |  |  | GAR-Cy2 (sNPF) |
| 5B | 2E |  | 0.1% OsO_4_ | GAR Au_12_ (sNPF) |
| 5C | 2E |  | 0.1% OsO_4_ | GAR Au_12_ (sNPF) |
| 6A | 1L |  |  | GAM-CY2 (AST-A) |
| 6B | 1L |  |  | GAR-CY3 (Proc) |
| 6C | 1L |  |  | Overlay of A and B |
| 6D | 1E |  | 0.5% OsO_4_ | GAR Au_6_ (AST-A)  GAM AU_12_ (Proc) |
| 7A | 2L |  |  | GAM-CY2 (AST-A) |
| 7B | 2L |  |  | GAR-CY3 (5-HT) |
| 7C | 2L |  |  | Overlay of A and B |
| 7D | 2E |  | 0.5% OsO_4_ | GAR Au_6_ (AST-A)  GAM AU_12_ (5-HT) |
| 8A | 1L |  |  | GAR-Cy2 (AST-A) |
| 8B | 1L |  |  | GAM-Cy3 (OA) |
| 8C | 1L |  |  | Overlay of A and B |
| 8D | 3E |  | 0.05% OsO_4_ | GAR Au_10_ (AST-A) |
| 8E | 3E |  | 0.05% OsO_4_ | PAP-DAB |
| 9A | 2L |  |  | GAM-Cy3 (AST-A) |
| 9B | 2L |  |  | GAR-Cy2 (sNPF) |
| 9C | 2L |  |  | Overlay of A and B |
| 9D | 2E |  | 0.1% OsO_4_ | GAR Au_12_ (sNPF) |
| 9E | 2E |  | 0.1% OsO_4_ | GAR Au_12_ (sNPF) |
| 9F | 2E |  | 0.1% OsO_4_ | GAR AU_12_ (AST-A) |
| 10A | 1L |  |  | GAR-CY2 (sNPF) |
| 10B | 1L |  |  | GAM-CY3 (OA) |
| 10C | 1L |  |  | Overlay of A and B |
| 10D | 1L |  |  | Overlay of A and B |
| 10E | 1L |  |  | Overlay of A and B |
| 11A | 2E |  | 0.1% OsO_4_ | GAR AU_12_ (sNPF) |
| 11B | 2E |  | 0.5% OsO_4_ | GAR AU_12_ (AST-A) |
| 11C | 2E |  | 0.5% OsO_4_ | GAR Au_6_- (PSK) |
| 11D | 2E |  | 0.1% OsO_4_ | GAR AU_12_ (LMS) |
