# Supplementary figures and images for: The complex neurochemistry of the cockroach antennal heart
Source: Cell Tissue Res. 2024 Sep 6;398(2):139–60. doi: 10.1007/s00441-024-03915-5 (PMC11525290; doi:10.1007/s00441-024-03915-5)

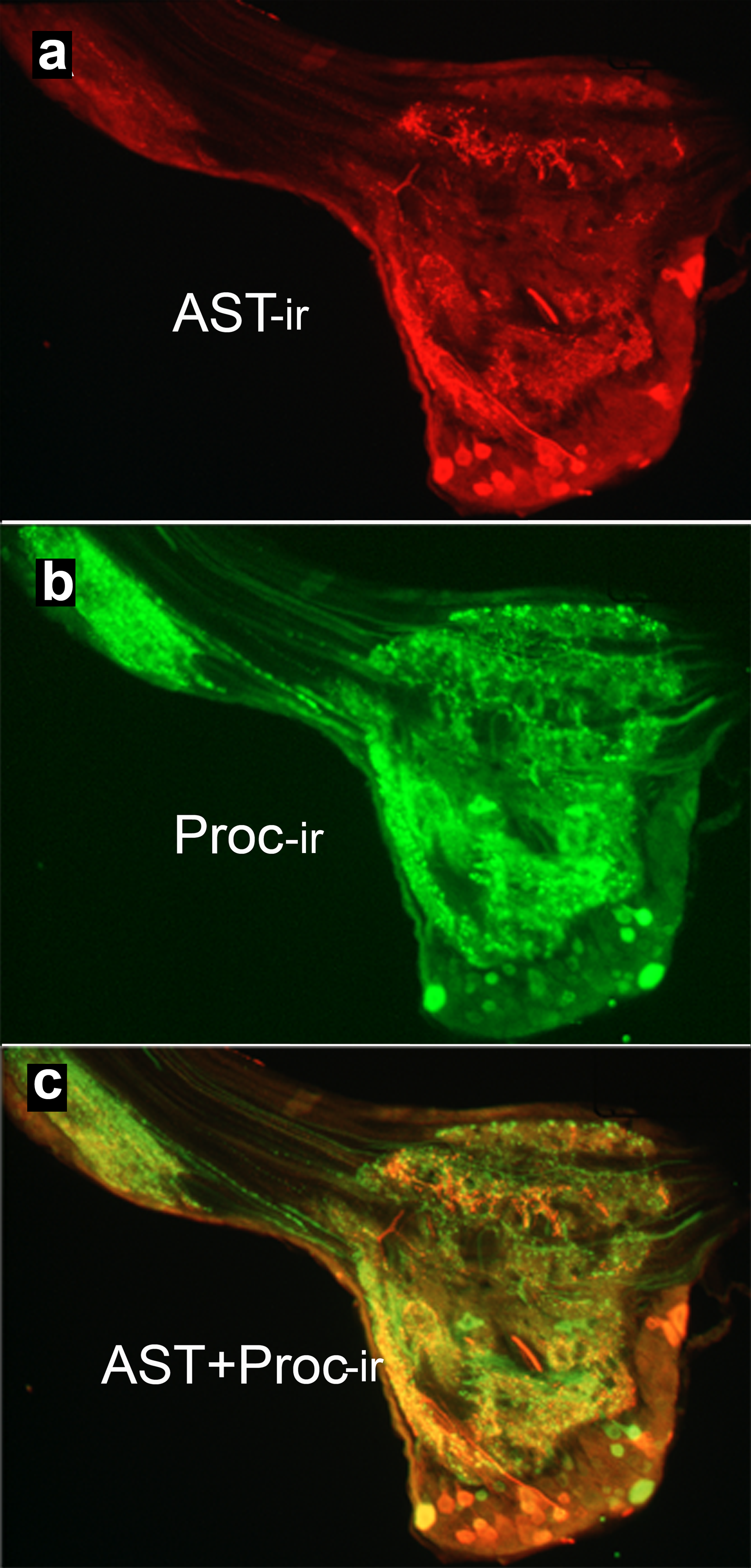

Supplement: Supplementary file 2 — Co-localization of allatostatin and proctolin in a single neuronal soma within the suboesophageal ganglion (SOG) A Parasagittal vibratome section of the SOG marked with AST A-ir (red). B The same section stained with PROC-ir (green). C Overlay of A and B shows that both antigens are present in a single soma (yellow). This soma is the only one within the SOG carrying both labels and corresponds in size and position the ClP soma described previously (Pass et al. 1988a). (PNG 3419 kb) [file 441_2024_3915_Fig13_ESM.png]

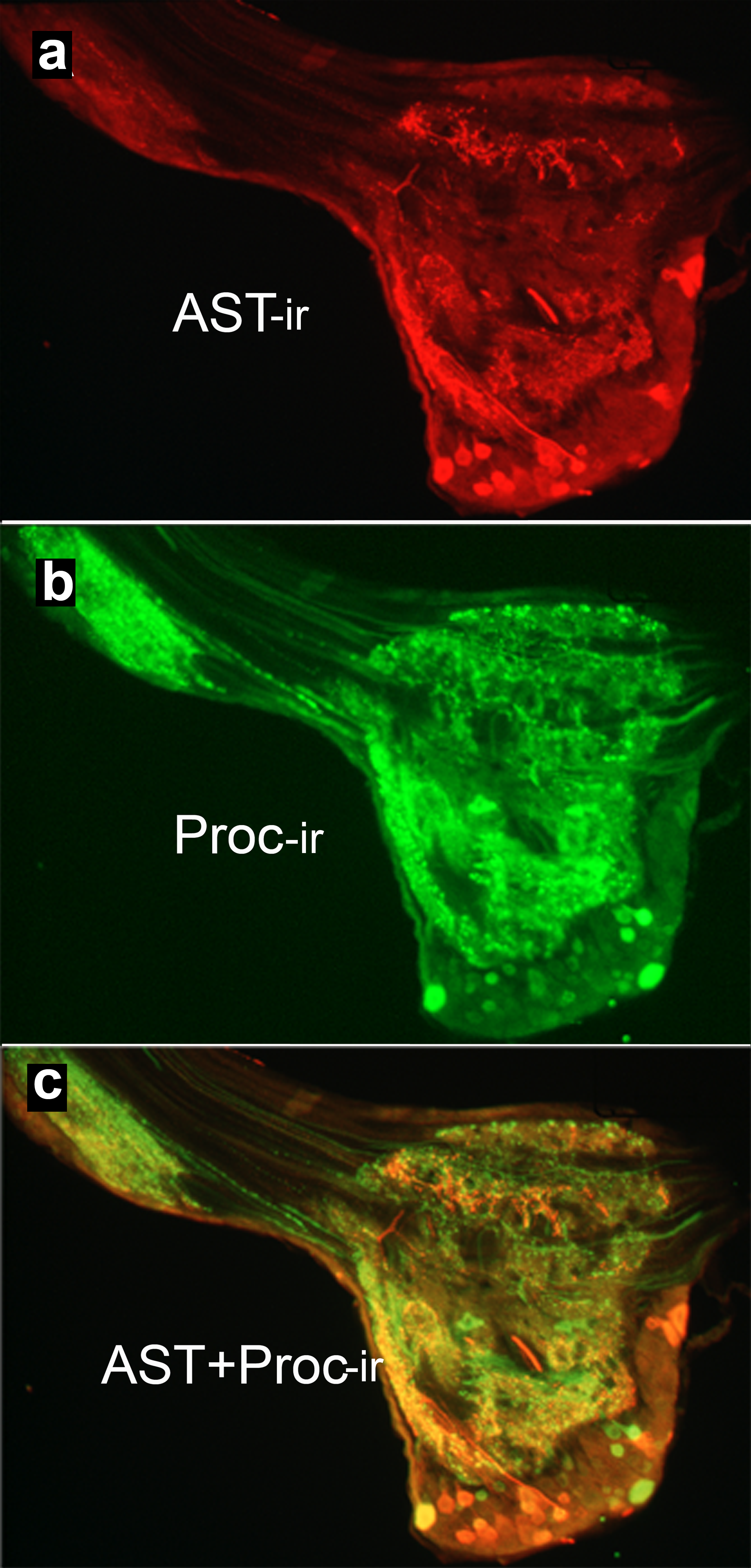

Supplement: Supplementary file 3 — High resolution image (TIF 29897 kb) [file 441_2024_3915_MOESM2_ESM.tif]

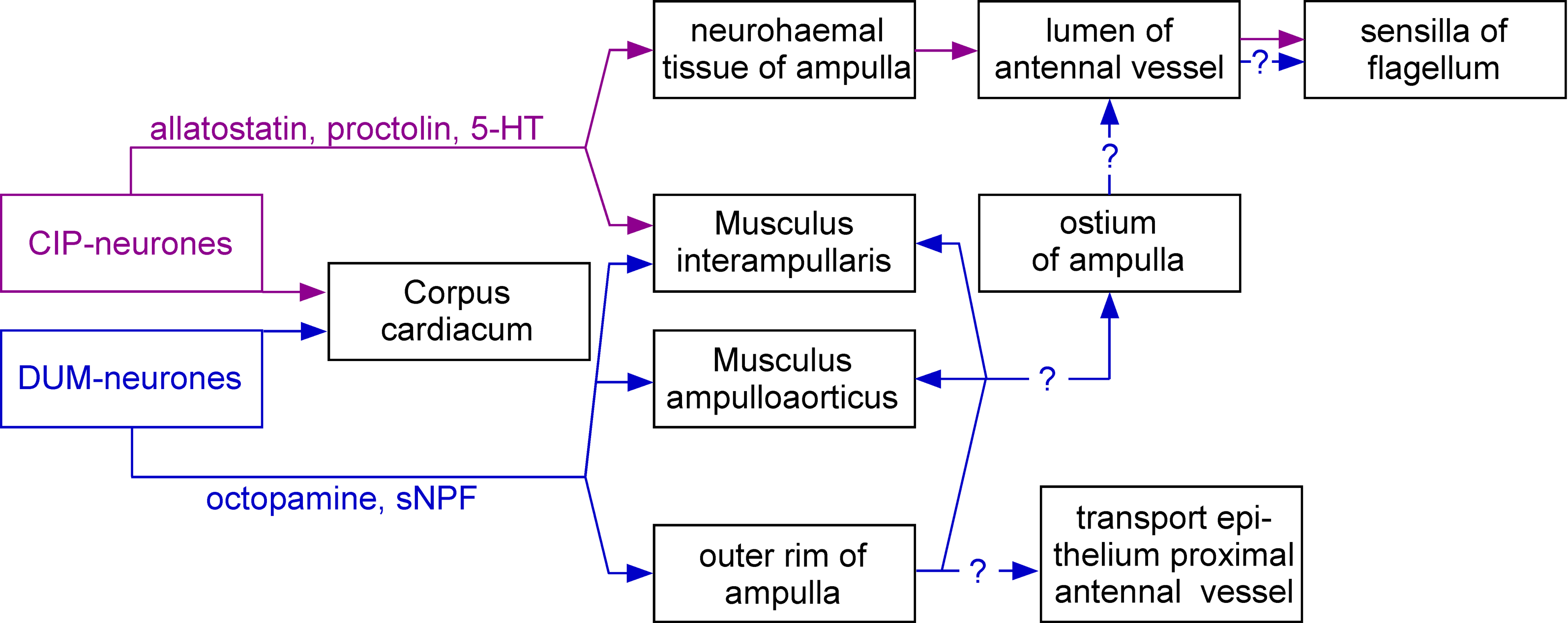

Supplement: Supplementary file 4 — Schematic representation of the pathways by which the neuromediators contained in the DUM and ClP neurons could reach substructures of the antennal heart and other targets. (PNG 226 kb) [file 441_2024_3915_Fig14_ESM.png]

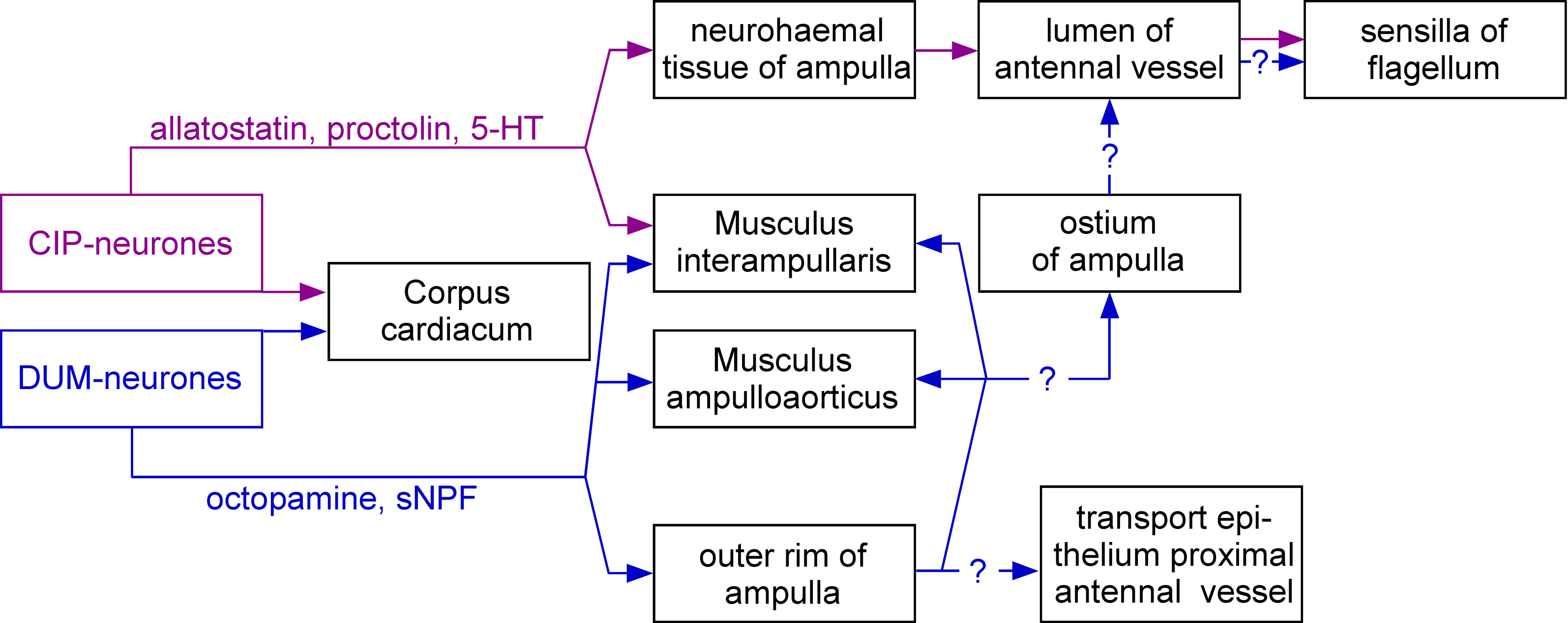

Supplement: Supplementary file 5 — High resolution image (TIF 11870 kb) [file 441_2024_3915_MOESM3_ESM.tif]

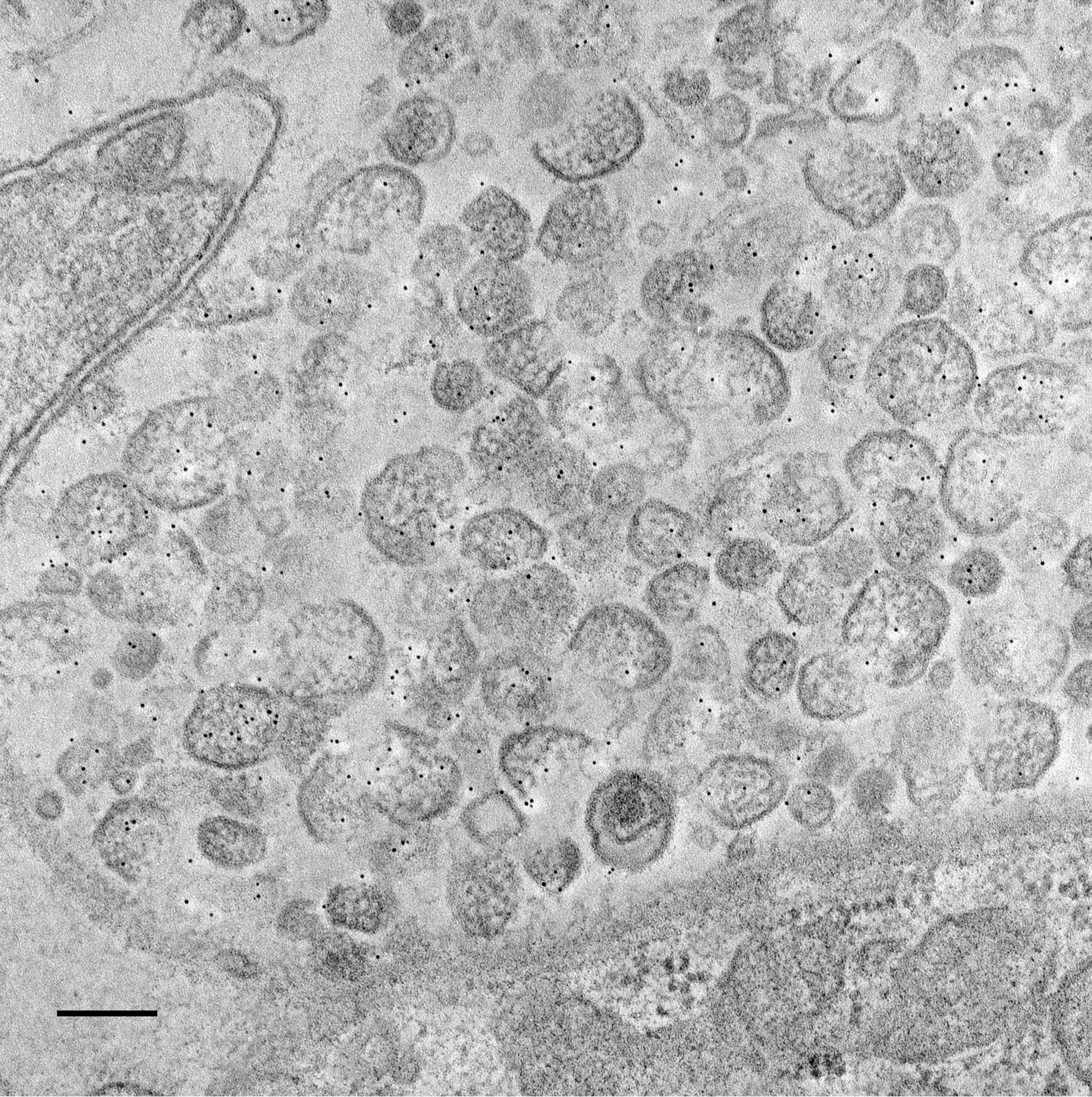

Supplement: Supplementary file 6 — Electron micrograph of an AST A-ir terminal of the ampulla. At high magnification and weak osmification (0.05% OsO4), the matrix of the neurosecretory granules appears vesicular. Scale bar: 155 nm (PNG 17493 kb) [file 441_2024_3915_Fig15_ESM.png]

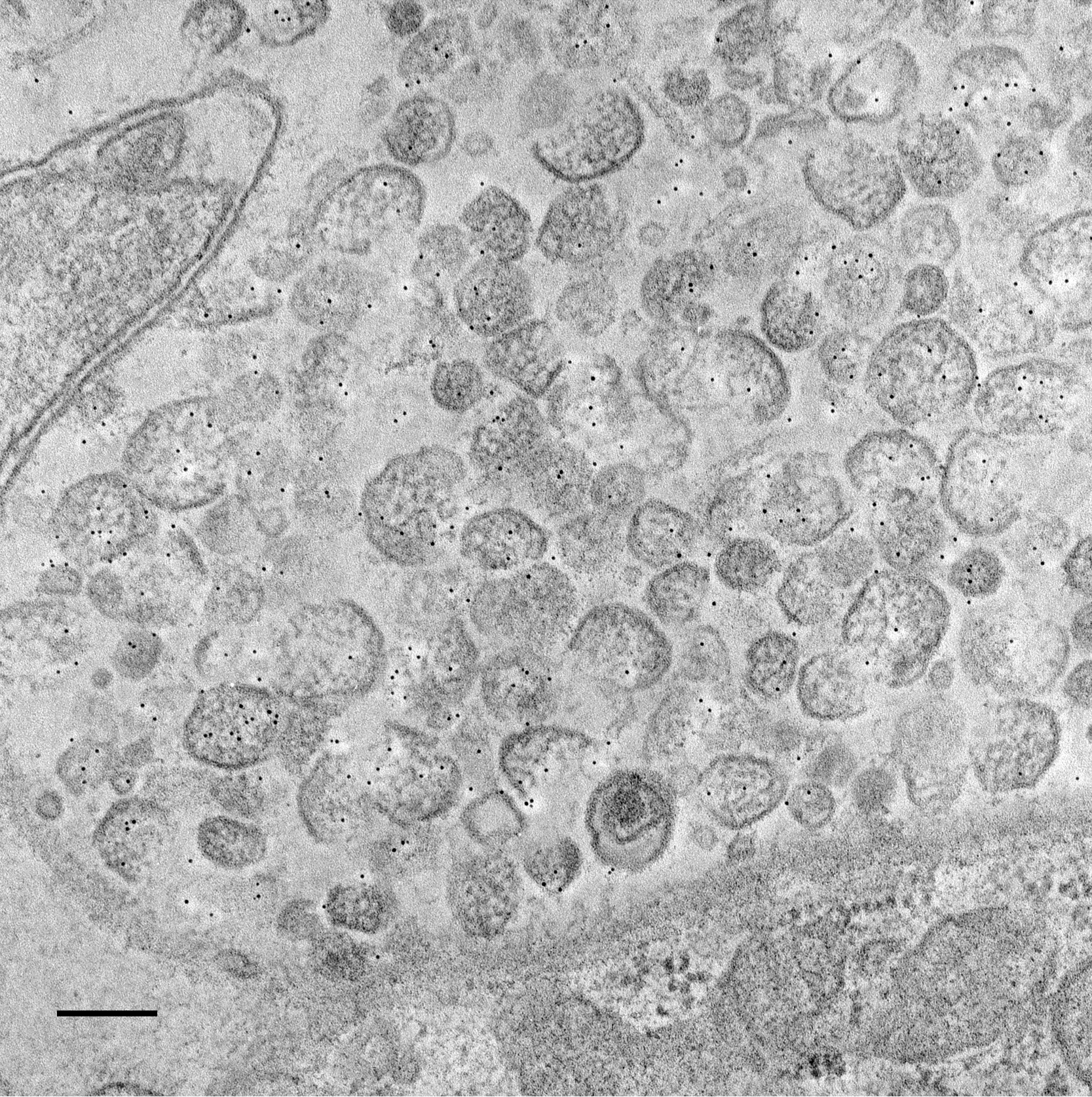

Supplement: Supplementary file 7 — High resolution image (TIF 2756 kb) [file 441_2024_3915_MOESM4_ESM.tif]

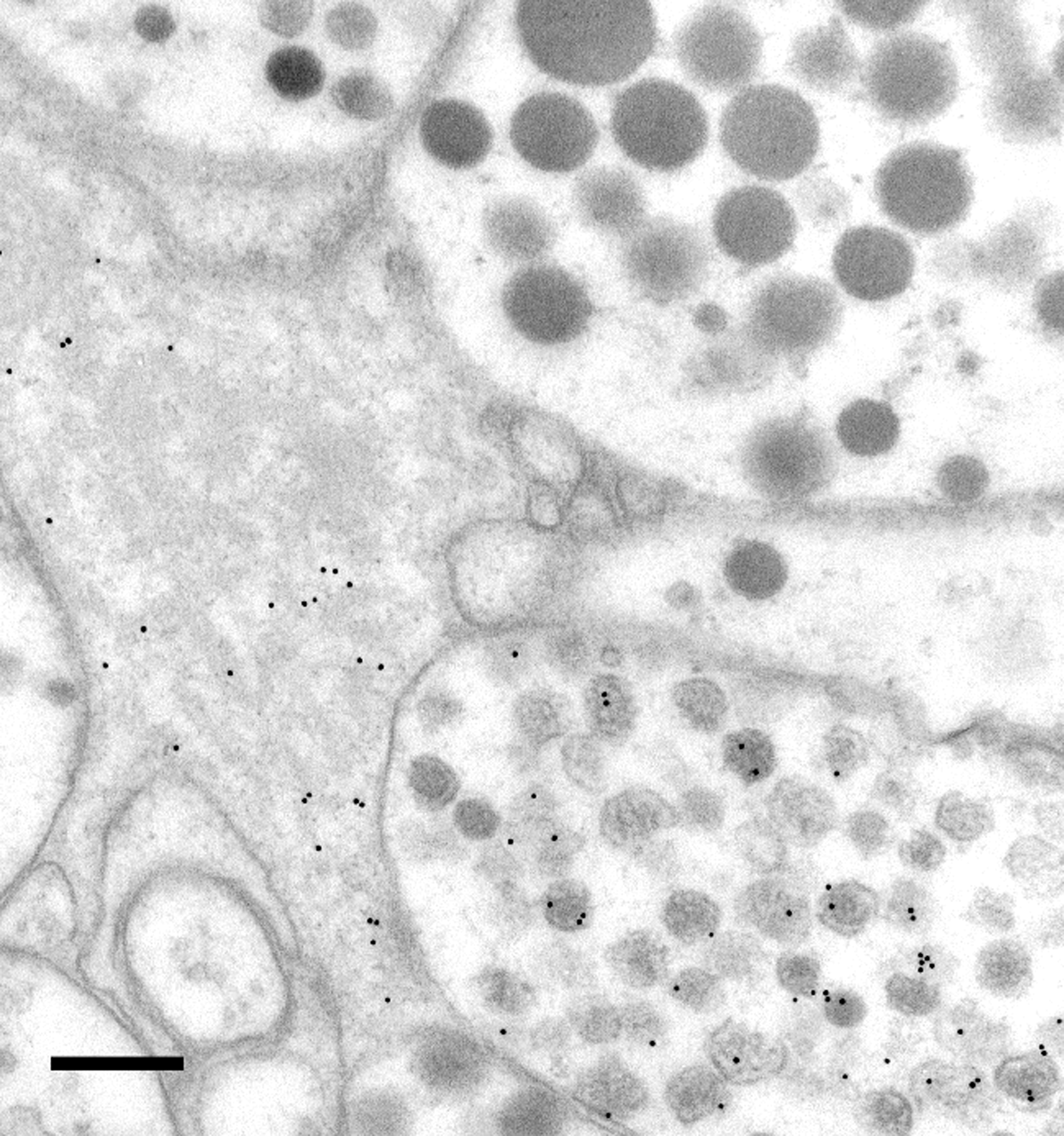

Supplement: Supplementary file 8 — Electron micrograph of an sNPF-ir terminal in the corpora cardiaca. Note the immu-noreactivity in the area of the basal lamina. Scale bar: 250 nm (PNG 1811 kb) [file 441_2024_3915_Fig16_ESM.png]

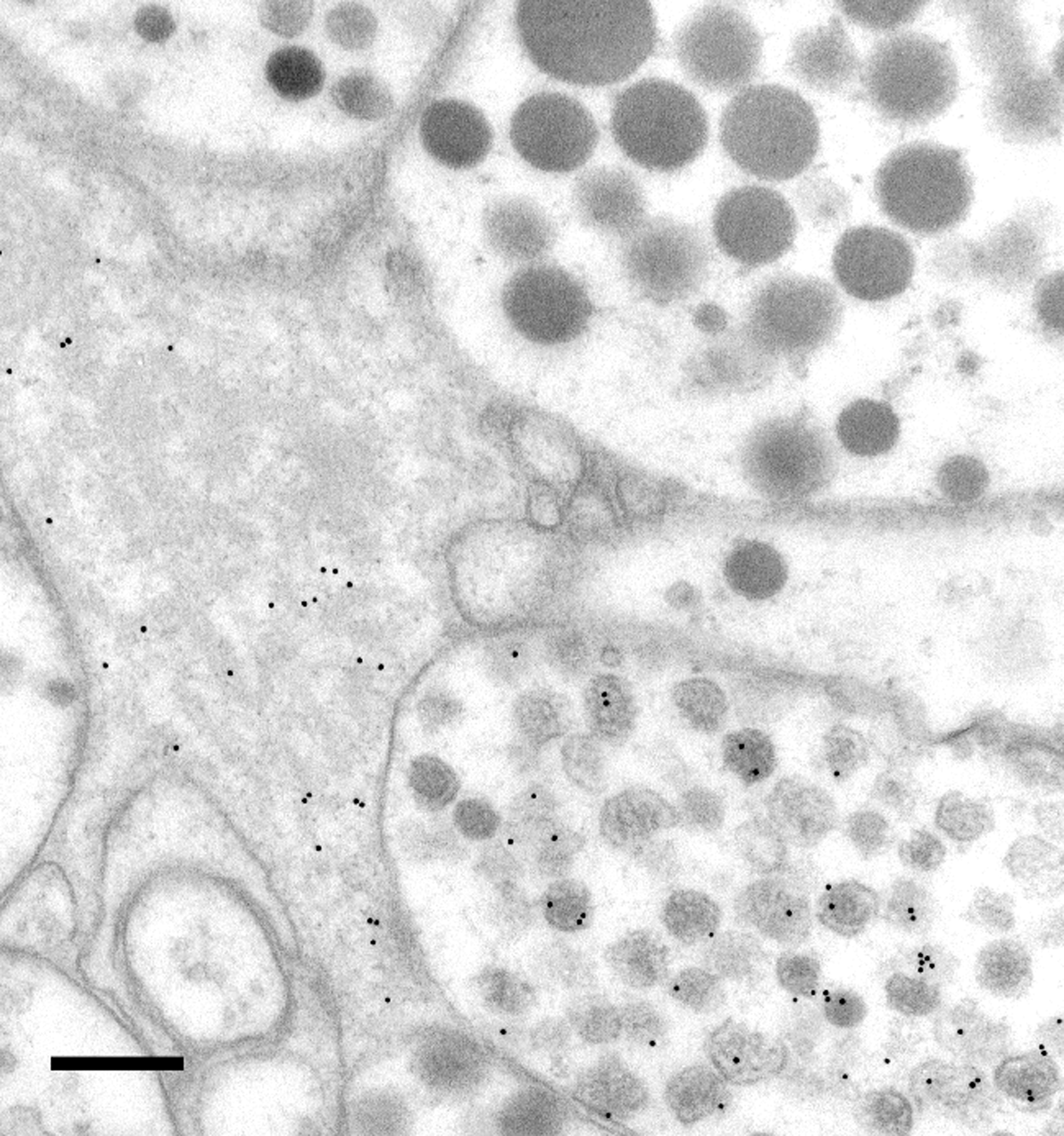

Supplement: Supplementary file 9 — High resolution image (TIF 16845 kb) [file 441_2024_3915_MOESM5_ESM.tif]
